# Continuous Suppression of Pathological Retinal and Choroidal Neovascularization in Cynomolgus Monkeys via Noninvasive Ophthalmic Delivery of a Novel Anti‐VEGFA Nanobody and Proprietary Penetratin Analog Formulation

**DOI:** 10.1002/advs.202504660

**Published:** 2025-10-21

**Authors:** Chong Chen, Min Zhu, Xingyan Fan, Kangjia Lv, Kuan Jiang, Yuchen Zhang, Haoliang Zhang, Xinming Qi, Benjamin TK Lee, Yakun Wan, Gang Wei, Kun Liu, Xun Xu

**Affiliations:** ^1^ Department of Ophthalmology, Shanghai General Hospital, School of Medicine, National Clinical Research Center for Eye Diseases, Shanghai Key Laboratory of Ocular Fundus Diseases Shanghai Jiao Tong University Shanghai 200080 China; ^2^ Shanghai Novamab Biopharmaceuticals Co., Ltd. Shanghai 201315 China; ^3^ Department of Pharmaceutics School of Pharmacy Key Laboratory of Smart Drug Delivery (Fudan University) Ministry of Education National Key Laboratory of Advanced Drug Formulations for Overcoming Delivery Barriers Fudan University Shanghai 201203 China; ^4^ Eye Institute and Department of Ophthalmology, Eye and ENT Hospital Fudan University Shanghai 200031 China; ^5^ Center for Drug Safety Evaluation and Research, State Key Laboratory of Drug Research, Shanghai Institute of Materia Medica Chinese Academy of Sciences Shanghai 201203 China; ^6^ Alephoson Biopharmaceuticals Limited Hong Kong SAR China; ^7^ The Institutes of Integrative Medicine of Fudan University Shanghai Engineering Research Center of ImmunoTherapeutics Shanghai 201203 China; ^8^ Quzhou Fudan Institute Quzhou 324003 China

**Keywords:** age‐related macular degeneration, cell‐penetrating peptides, diabetic retinopathy, nanobody, neovascularization, noninvasive intraocular delivery, single‐domain antibody

## Abstract

Pathological retinal and choroidal neovascularization is a hallmark of several blinding diseases, including diabetic retinopathy and age‐related macular degeneration. While intravitreal anti‐VEGF therapies remain the standard of care, they necessitate frequent injections, posing risks such as endophthalmitis and elevated intraocular pressure, alongside economic and adherence challenges. Here, we present Pene/LQ015, a novel eye drop formulation comprising the anti‐VEGFA nanobody (LQ015) and a proprietary penetratin analog for noninvasive delivery. LQ015 demonstrates superior VEGF‐blocking activity, broad binding specificity across species, and robust stability and scalability using a yeast expression system. Topical administration of Pene/LQ015 achieved effective retinal‐choroid complex drug levels and suppressed neovascularization in preclinical models. Notably, in the cynomolgus monkey laser‐induced choroidal neovascularization model, 30 days of continuous topical application significantly reduced neovascularization and vascular leakage, with excellent safety and tolerability. These findings highlight Pene/LQ015's potential as a game‐changer in treating neovascular eye diseases. It offers a groundbreaking, noninvasive alternative to intravitreal injections, addressing key limitations of current therapies by enabling continuous dosing, improving patient adherence, and reducing treatment burden. These findings underscore its potential to transform the management of neovascular retinal and choroidal diseases, with promising implications for clinical application.

## Graphical abstract

Pathological retinal/choroidal neovascularization drives vision loss in DR and AMD. Current anti‐VEGF injections, while effective, require invasive, frequent administration. Pene/LQ015, is developed a novel topical anti‐VEGFA nanobody formulation achieving therapeutic intraocular levels through penetratin analog‐enhanced delivery. Preclinical studies in primate CNV models showed significant neovascularization suppression with safety, positioning this noninvasive treatment as a paradigm‐shifting alternative for fundus angiogenesis diseases.

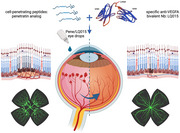

## 1 Introduction

Pathological retinal and choroidal neovascularization underlies the pathogenesis of major vision‐threatening diseases, including diabetic retinopathy,^[^ ^1^, ^2^ ^]^ age‐related macular degeneration,^[^ ^3^, ^4^ ^]^ and retinal vein occlusion,^[^ ^5^, ^6^ ^]^ collectively representing leading causes of irreversible blindness worldwide. The current standard of care relies on intravitreal administration of anti‐vascular endothelial growth factor (VEGF) agents, which has revolutionized the management of these conditions and associated macular edema.^[^ ^2^, ^4^, ^6^, ^7^ ^]^ However, this therapeutic paradigm presents inherent limitations: the invasive nature of repeated intraocular injections carries risks of complications such as endophthalmitis, intraocular inflammation, vitreous hemorrhage, cataract formation, and elevated intraocular pressure.^[^ ^4^, ^8^, ^9^ ^]^ Moreover, the demanding treatment regimen imposes considerable economic burdens, compromises patient compliance, and ultimately leads to suboptimal real‐world outcomes.^[^ ^4^ ^]^

The development of effective non‐invasive therapies faces two major pharmacological challenges. Macromolecular anti‐VEGF drugs (48–150 kDa) exhibit poor penetration through ocular biological barriers when administered topically.^[^ ^10^, ^11^, ^12^ ^]^ Conversely, small molecular biological peptides (2–4 kDa), while demonstrating better tissue penetration, are hampered by intrinsic limitations including poor stability, insufficient potency, and a short biological half‐life, which leads to rapid clearance from the body and limits sustained therapeutic effects.^[^ ^13^ ^]^

Nanobodies (Nbs), the recombinant variable domains of heavy chain‐only antibodies (VHH) derived from Camelidae species, have emerged as a promising therapeutic platform.^[^ ^14^, ^15^, ^16^, ^17^ ^]^ These nanoscale antibodies (2.5 × 4 nm) combine unique pharmacological properties, including exceptional stability, high target affinity and specificity, superior solubility, access to cryptic epitopes, enhanced tissue penetration, and scalable production.^[^ ^18^, ^19^ ^]^ As the smallest known functional antibody fragments in nature,^[^ ^14^ ^]^ Nbs have emerged as a novel class of therapeutics, distinct from conventional antibodies and offering unique advantages for ocular therapeutics.

To overcome ocular barrier limitations, we have incorporated cell‐penetrating peptides (CPPs) into our therapeutic strategy.^[^ ^20^ ^]^ CPPs are small bioactive peptides typically composed of fewer than 30 amino acids that demonstrate remarkable membrane permeability.^[^ ^20^, ^21^, ^22^, ^23^ ^]^ These versatile peptide carriers can effectively facilitate the cellular uptake of various therapeutic compounds through either covalent conjugation or non‐covalent complexation, while maintaining excellent biocompatibility and minimal cytotoxic effects.^[^ ^21^ ^]^ CPPs, particularly penetratin, have demonstrated remarkable capacity to facilitate the transport of therapeutic cargo across biological barriers, including the blood‐ocular barrier,^[^ ^20^, ^24^ ^]^ blood‐brain barrier,^[^ ^25^ ^]^ and even intratumoral barriers.^[^ ^26^ ^]^ Penetratin, characterized by its strong positive net charge (+8.0), has shown particular promise as an absorption enhancer for noninvasive intraocular drug delivery.^[^ ^20^, ^24^ ^]^ Its transcytosis mechanism involves both direct transmembrane transport, mediated by electrostatic interactions with negatively charged cell membranes, and endocytic pathways through caveolae and clathrin‐mediated processes.^[^ ^27^, ^28^ ^]^

Building upon these scientific advances, we have developed Pene/LQ015, an innovative eye drop formulation that represents a potential paradigm shift in the treatment of neovascular eye diseases. This novel therapeutic eye drop combines two key components: 1) LQ015, a next‐generation anti‐VEGFA nanobody engineered for superior target engagement, and 2) a proprietary penetratin analog (sequence: RWIKIWFWWRRMKWKK), specifically designed to facilitate efficient ocular barrier penetration while maintaining optimal safety and efficacy profiles. This combination harnesses the unique advantages of both nanobody technology and advanced peptide‐mediated drug delivery, offering a promising non‐invasive alternative to current intravitreal therapies.

## 2 Results

### 2.1 Nb Library Screening for Functional Nbs – Converges on One Dominant Nb20

**Figure 1:**
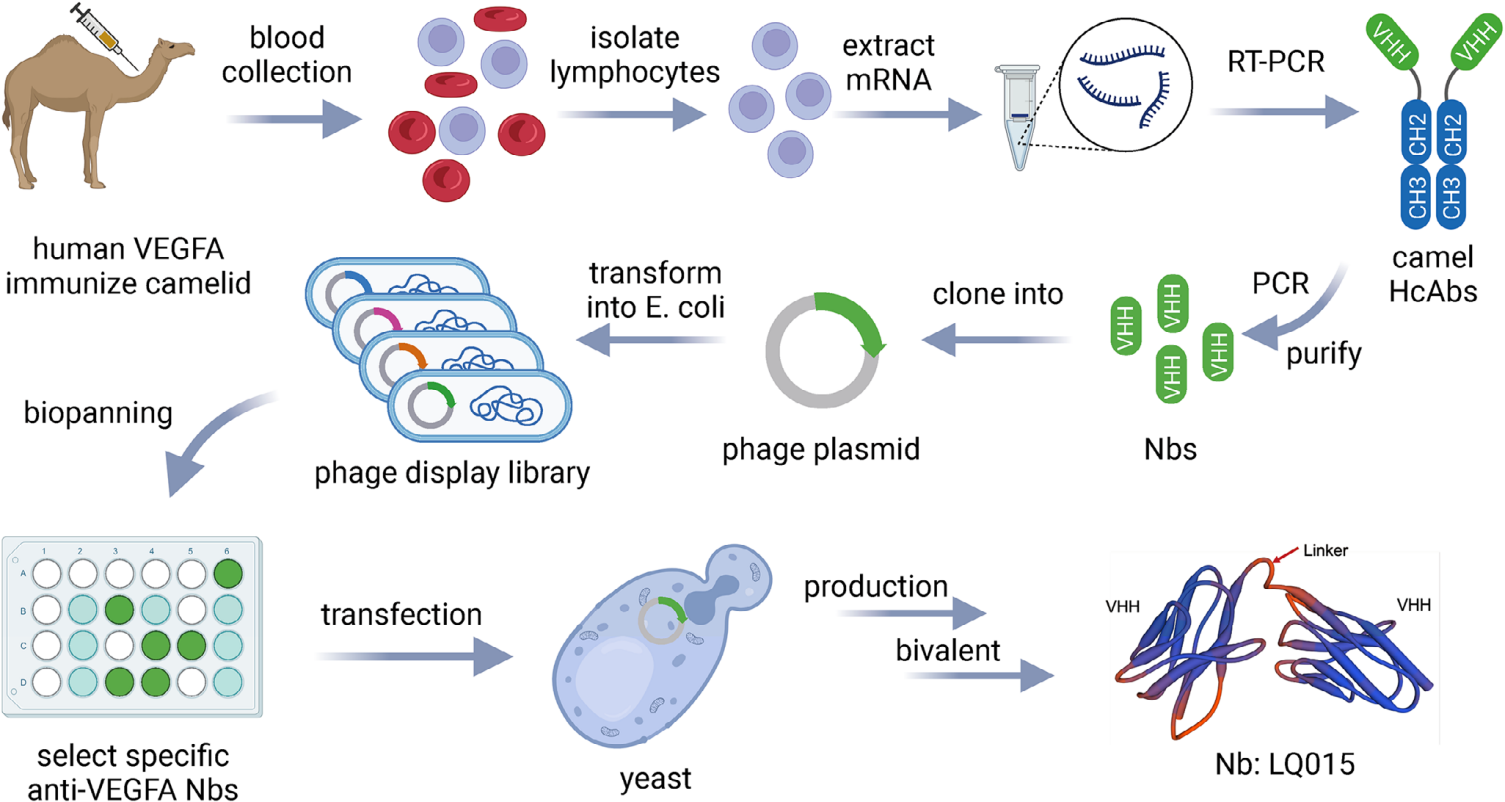
The schematic diagram of the production and synthesis of bivalent specific anti‐VEGFA nanobodies (Nb), created by BioRender.com.

**Figure 2:**
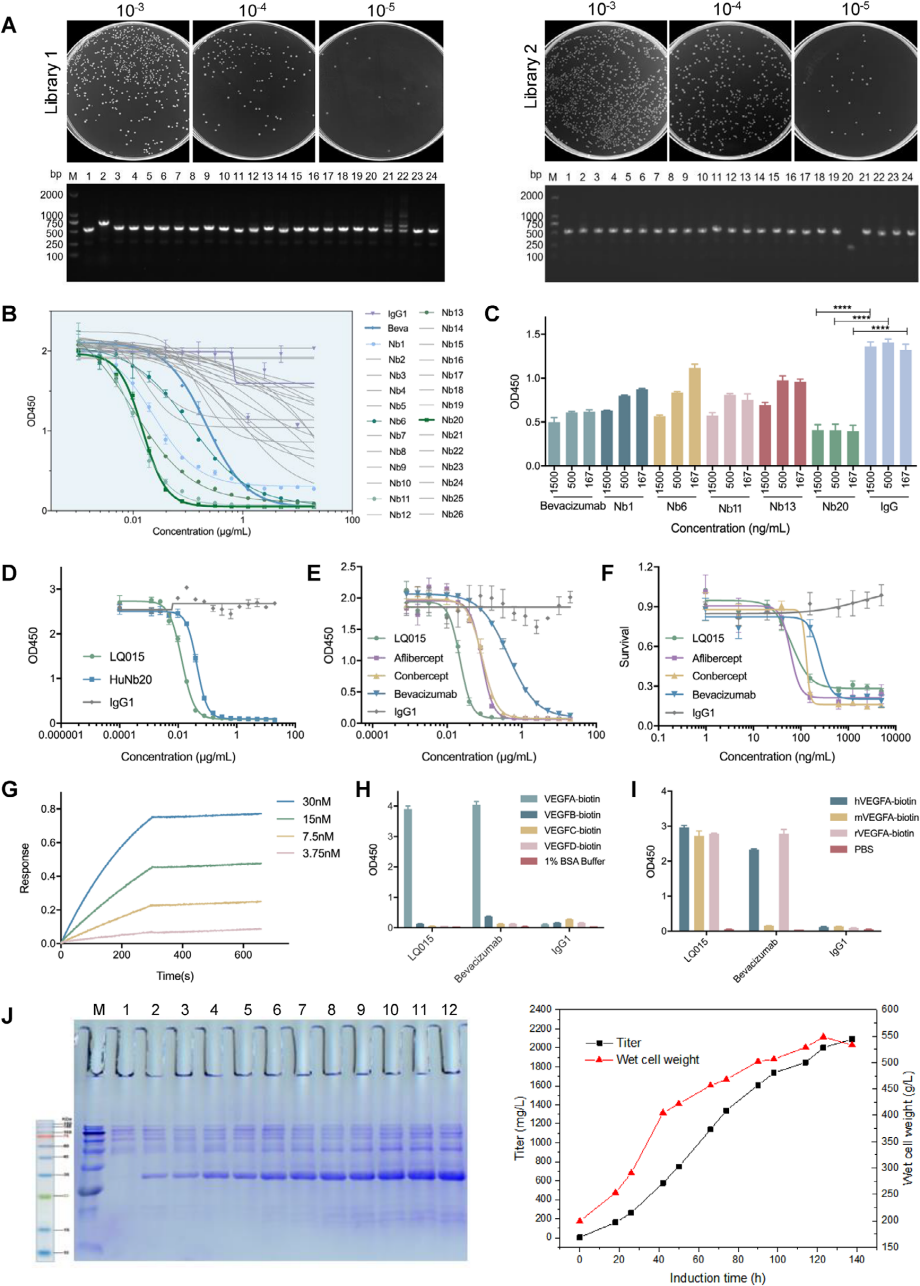
Functional Nb candidates Screening and large‐scale production of LQ015. A) Nb library construction, gradient dilution, and volume determination. The 24 clones were randomly selected from the gradient dilution plates for colony polymerase chain reaction to detect the insertion rate of the nanobody libraries. B) The blocking activity of VEGF binding to VEGFR2 was assessed using ELISA, with Nb20 demonstrating significantly superior blocking activity compared to the control antibody Bevacizumab (IC_50 Nb20_ = 0.015 µg mL^−1^, IC_50 Bevacizumab_ = 0.22 µg mL^−1^). C) Nb20 demonstrated significantly enhanced inhibitory activity against HUVEC proliferation compared to Bevacizumab (IC_50 Nb20_ = 29.58 ng mL^−1^, IC_50 Bevacizumab_ = 72.28 ng mL^−1^). D) The bivalent humanized Nb20 (LQ015) exhibited superior hVEGFA/hVEGFR2 blocking activity compared to the monomer HuNb20. E) LQ015 demonstrates significantly superior blocking activity in comparison to commonly utilized anti‐VEGF agents in clinical practice, as evidenced by its lower IC_50_ value (0.022 µg mL^−1^) compared to Aflibercept (0.085 µg mL^−1^), Conbercept (0.088 µg mL^−1^), and Bevacizumab (0.44 µg mL^−1^). F) LQ015 exhibits a more potent inhibitory effect on the proliferation of HUVECs when compared to other anti‐VEGF therapeutics. G) LQ015 showed strong affinity with hVEGFA (Kd = 1 x 10^−12^ M). H) LQ015 was specifically bound to hVEGFA without cross reaction with other proteins of the same family, such as hVEGFB, hVEGFC, and hVEGFD. I) LQ015 could recognize not only human VEGFA, but also rabbit VEGFA and mouse VEGFA. J) The protein expression of LQ015 was initially analyzed using SDS‐PAGE, followed by purification steps including anion chromatography and hydrophobic chromatography. The protein titer and wet cell weight in the fermentation tank were monitored at specified intervals. All ELISA measurements were performed in duplicate (*n* = 2), and cell‐based experiments were repeated at least three times independently (n ≥ 3). Data are presented as means ± SEM in (B‐F, H, I) and analyzed with one‐way ANOVA, followed by Tukey's multiple comparison in (C). ^****^ *p* < 0.0001.

**Figure** **1** illustrates the comprehensive screening workflow for anti‐VEGFA Nbs. Initial library construction yielded high‐capacity VHH libraries with 6.4 × 10^8^ and 5.5 × 10^8^ colony‐forming units, with insertion rates of 91.7% and 95.8%, respectively (**Figure** **2**A). Subsequent phage display screening involved six and five iterative rounds of biopanning for phage enrichment, as detailed in Figure S1A (Supporting Information). Following library construction, high‐throughput screening of 300 clones via enzyme‐linked immunosorbent assay (ELISA) identified over 80 positive candidates (Figure S1B, Supporting Information), which were subsequently sequenced and classified into 26 distinct phylogenetic families.

Comparative functional analysis revealed five lead candidates (Nb1, Nb6, Nb11, Nb13, and Nb20) demonstrating superior VEGFA blocking activity compared to the clinical benchmark Bevacizumab (Avastin) (Figure 2B). Among these, Nb20 emerged as the most potent inhibitor, exhibiting remarkable blocking efficacy with an IC_50_ of 0.015 µg mL^−1^, representing a 14.7‐fold improvement over Bevacizumab (IC_50_ = 0.22 µg mL^−1^). This enhanced pharmacological activity was further corroborated by Nb20's significantly greater inhibition of human umbilical vein endothelial cells (HUVECs) proliferation compared to Bevacizumab (*p* < 0.0001, Figure 2C).

To optimize the therapeutic potential of Nb20, we engineered a bivalent humanized derivative (designated LQ015) using a yeast expression system. Structural optimization enabled LQ015 to exhibit enhanced hVEGFA/hVEGFR2 blocking activity compared to its monomeric counterpart (Figure 2D), indicative of improved target engagement and therapeutic efficacy.

### 2.2 Molecular Biological Activity of LQ015

Furthermore, LQ015 demonstrated markedly superior pharmacological activity compared to clinically established anti‐VEGF agents, as quantified through comprehensive bioassays. The IC_50_ analysis revealed that LQ015 (0.022 µg mL^−1^) exhibited 3.9‐fold, 4.0‐fold, and 20‐fold greater potency than Aflibercept (Eylea, 0.085 µg mL^−1^), Conbercept (Lumitin, 0.088 µg mL^−1^), and Bevacizumab (0.44 µg mL^−1^), respectively (Figure 2E). Consistent with these findings, HUVEC proliferation assays confirmed LQ015's enhanced inhibitory capacity relative to other anti‐VEGF therapeutics (Figure 2F).

Binding affinity studies demonstrated LQ015's strong affinity and high specificity for hVEGFA, with no detectable cross‐reactivity to other VEGF family members (hVEGFB, hVEGFC, and hVEGFD) (Figure 2G,H). Notably, LQ015 exhibited cross‐species recognition capability, effectively binding to VEGFA from human, mouse, and rabbit sources (Figure 2I). Functional assays revealed potent inhibition of VEGF/VEGFR2 interactions across multiple species, with IC50 values of 0.026 µg mL^−1^ in rabbits, 0.070 µg mL^−1^ in rats, and 0.0036 µg mL^−1^ in mice (Figure S1C–E, Supporting Information). These findings highlight the cross‐species efficacy of LQ015 and support its applicability in preclinical studies.

### 2.3 Large‐Scale Production and Stability of LQ015

**Figure 3:**
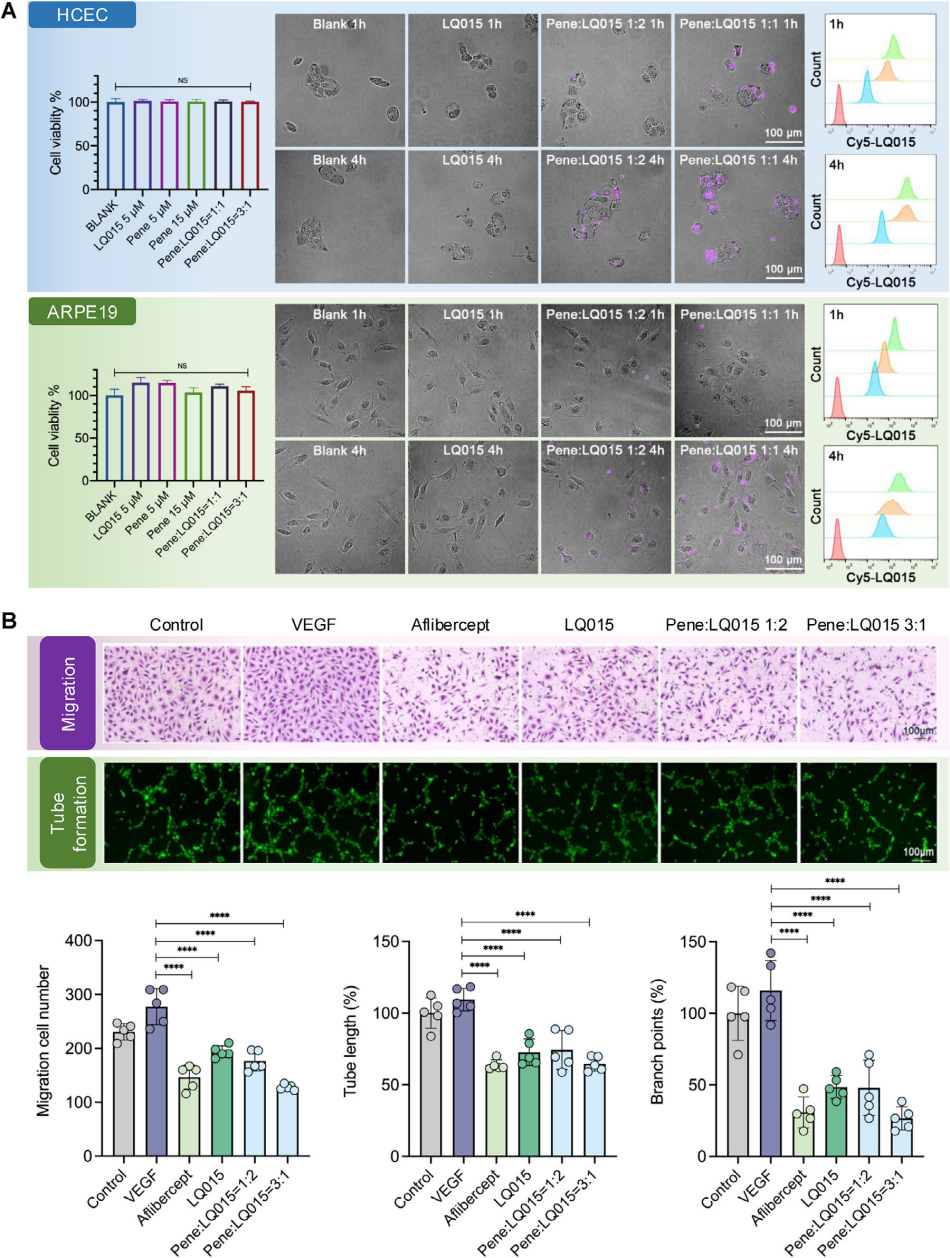
A) Pene/LQ015 eye drops (1:1, 3:1 ratio) had no obvious cytotoxicity on the growth of HCEC and ARPE19 cells (left, *n* = 3). Analysis of intracellular uptake evaluated by high‐content imaging (middle) and flow cytometry (right, *n* = 3) at 1 and 4 h posttreatment. Flow cytometry profiles showing cellular internalization: untreated control (red), LQ015 alone (blue), Pene:LQ015 (1:2, orange), and Pene:LQ015 (3:1, green). Data demonstrate enhanced cellular penetration of Pene/LQ015 formulations compared to LQ015 alone. B) Anti‐angiogenic effects of LQ015 and Pene/LQ015 in HRMECs (*n* = 5). Both LQ015 and Pene/LQ015 formulations (at 1:2 and 3:1 ratios) demonstrated significant inhibition of VEGF‐induced migration and tube formation in HRMECs (*p* < 0.0001 vs VEGF). The anti‐angiogenic efficacy was comparable to Aflibercept (*p* > 0.05). Data are presented as means ± SEM and analyzed with one‐way ANOVA, followed by Tukey's multiple comparison in (A, B). NS, not significant, *p* > 0.05; ^****^ *p* < 0.0001.

Scale‐up production of LQ015 using a yeast expression system^[^ ^15^, ^16^ ^]^ achieved robust yields and purity. The fermentation process yielded 500 g L^−1^ wet cell weight and 2.0 g L^−1^ protein after 120 h in a 100 L bioreactor (**Figure** **3**A). Purification through affinity, hydrophobic interaction, and size‐exclusion chromatography yielded high‐purity product, as confirmed by high‐performance liquid chromatography analysis. Stability assessments demonstrated that LQ015 maintained structural integrity for 30 days at 25 °C and 15 days at 40 °C (Figure S1F, Supporting Information), indicating favorable thermal stability for therapeutic applications.

### 2.4 In Vitro Cytotoxicity and Penetration of Pene/LQ015 Compounds at Different Molar Ratio

Despite the promising molecular activities of LQ015, the noninvasive delivery of biologics to the posterior segment of the eye, particularly the vitreous and retina‐choroid complex, remains a significant challenge due to multiple ocular barriers, including the blood‐retinal barrier and the functional blood‐aqueous barrier.^[^ ^10^, ^29^, ^30^ ^]^ To address this limitation, we developed a novel eye drop formulation by mixing negatively charged LQ015 with a positively charged cell‐penetrating peptide penetratin analog through non‐covalent electrostatic interactions,^[^ ^21^ ^]^ designed to facilitate the noninvasive delivery of LQ015 to the retina‐choroid complex.

To optimize the formulation and characterize its permeability, we employed high‐content imaging and flow cytometry to assess intracellular uptake in human corneal epithelial cells (HCEC) and human retinal pigment epithelium‐19 (ARPE19) cells. Our results demonstrated that Pene/LQ015 eye drops at 3:1 molar ratio showed no significant cytotoxicity while effectively enhancing LQ015 penetration into both HCEC and ARPE19 cells, in contrast to the limited cellular penetration observed with LQ015 alone (Figure 3C).

### 2.5 In Vitro Antiangiogenic Effects of LQ015 and Pene/LQ015 Compounds in HRMECs

As shown in Figure 3B, LQ015 and Pene/LQ015 (1:2 and 3:1) significantly suppressed VEGF‐stimulated Human Retinal Microvascular Endothelial Cells (HRMECs) migration and tube formation (*p* < 0.0001 vs VEGF), with efficacy comparable to Aflibercept (*p* > 0.05).

### 2.6 In Vivo Intravitreal Injection of LQ015 Showed Antiangiogenesis Efficacy and Safety in Rabbits and Rhesus Monkeys

**Figure 4:**
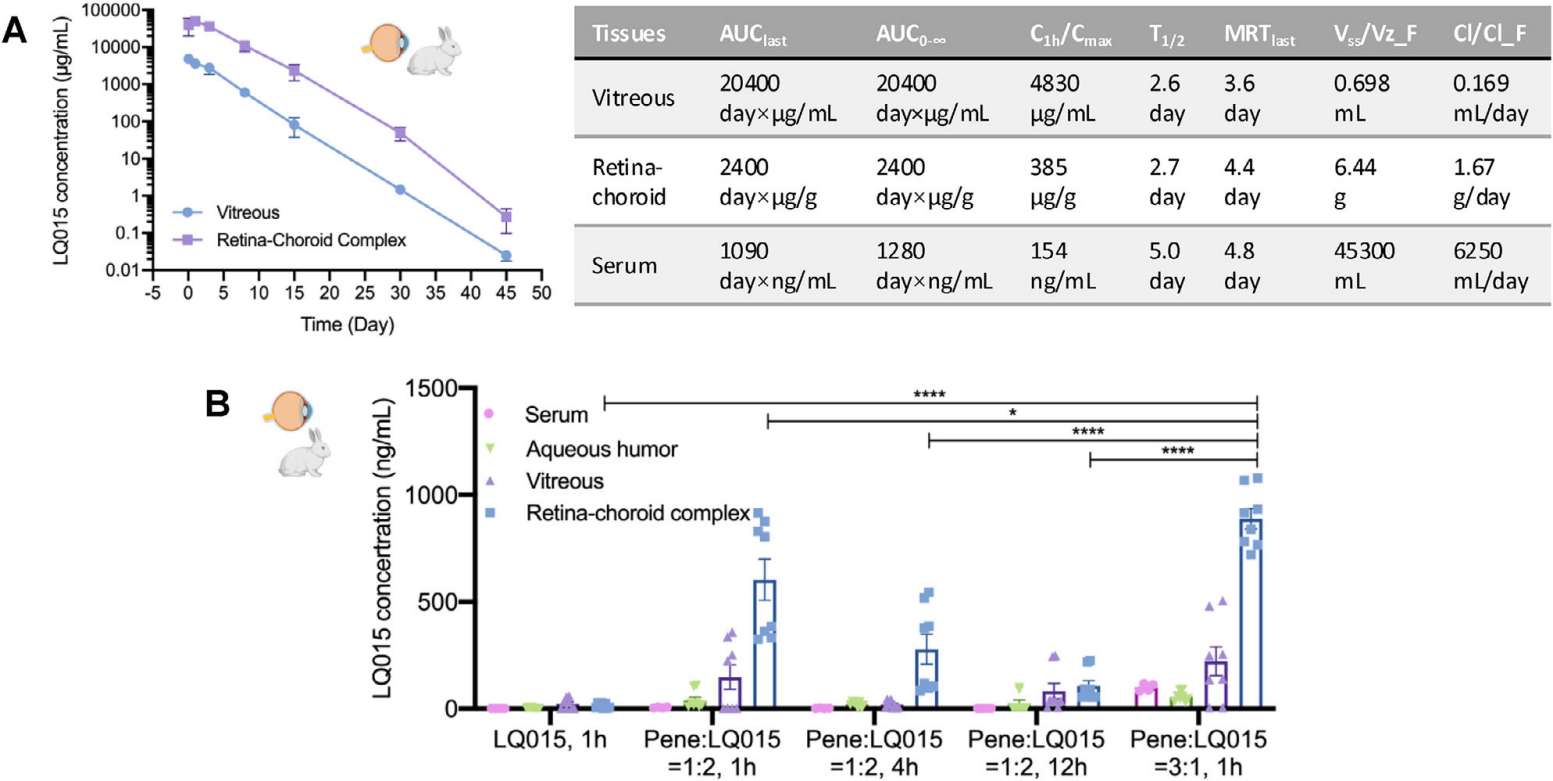
A) Pharmacokinetic analysis of LQ015 following intravitreal injection in rabbits showed a half‐life (T_1/2_) of 2.6 days in the vitreous and 2.7 days in the retina–choroid complex (*n* = 6). Key pharmacokinetic parameters of LQ015 in serum and ocular tissues are summarized. B) A pharmacodynamic study comparing topical administration of LQ015 alone versus various molar ratios of Penetratin analog and LQ015 was conducted in rabbits (serum: *n* = 4; ocular tissues/humors: *n* = 8). Results demonstrated significantly enhanced LQ015 absorption in the retina–choroid complex with the 3:1 Pene/LQ015 formulation at 1 h (887.8 ± 135.2 ng mL^−1^), compared to other treatment groups (*p* < 0.05). AUC, area under the curve; MRT, mean residence time; T_1/2_, half‐life; Cl, clearance. Data are presented as means ± SEM in (A, B) and analyzed with one‐way ANOVA, followed by Tukey's multiple comparison in (B). ^*^ *p* < 0.05; ^****^ *p* < 0.0001.

Intravitreal administration studies in rabbits established LQ015's favorable safety profile (Figure S2A,B, Supporting Information) and pharmacokinetic characteristics. The elimination half‐life (T_1/2_) was determined to be 2‐3 days in both vitreous humor and retina‐choroid complex following single‐dose administration (**Figure** **4**A).

Importantly, intravitreal LQ015 injection demonstrated significant efficacy in inhibiting laser‐induced choroidal neovascularization in rhesus monkeys, as evidenced by comprehensive in vivo assessments (Figure S2C–I, Supporting Information).

### 2.7 In Vivo Pharmacodynamic and Tissue Distribution Study of Pene/LQ015 Eye Drops in Rabbits

Pharmacokinetic evaluation in rabbit eyes demonstrated that the Pene/LQ015 eye drops (3:1 ratio, 50 µL, LQ015 40 mg mL^−1^) achieved therapeutically relevant concentrations in the retina–choroid complex, indicating efficient posterior segment delivery. Following topical administration (3 times daily for 7 consecutive days), the 3:1 formulation yielded significantly higher LQ015 concentrations in the retina‐choroid complex (887.8 ± 135.2 ng mL^−1^ at 1 h) compared to both LQ015 alone (14.00 ± 11.99 ng mL^−1^, *p *< 0.0001) and the 1:2 formulation (603.5 ± 272.7 ng mL^−1^ at 1 h, *p* < 0.05; 278.5 ± 198.7 ng mL^−1^ at 4 h, *p* < 0.0001; 105.2 ± 73.42 ng mL^−1^ at 12 h, *p* < 0.0001) (Figure 4B).

### 2.8 In Vivo Efficacy Study of Pene/LQ015 Eye Drops in Mouse OIR Model

**Figure 5:**
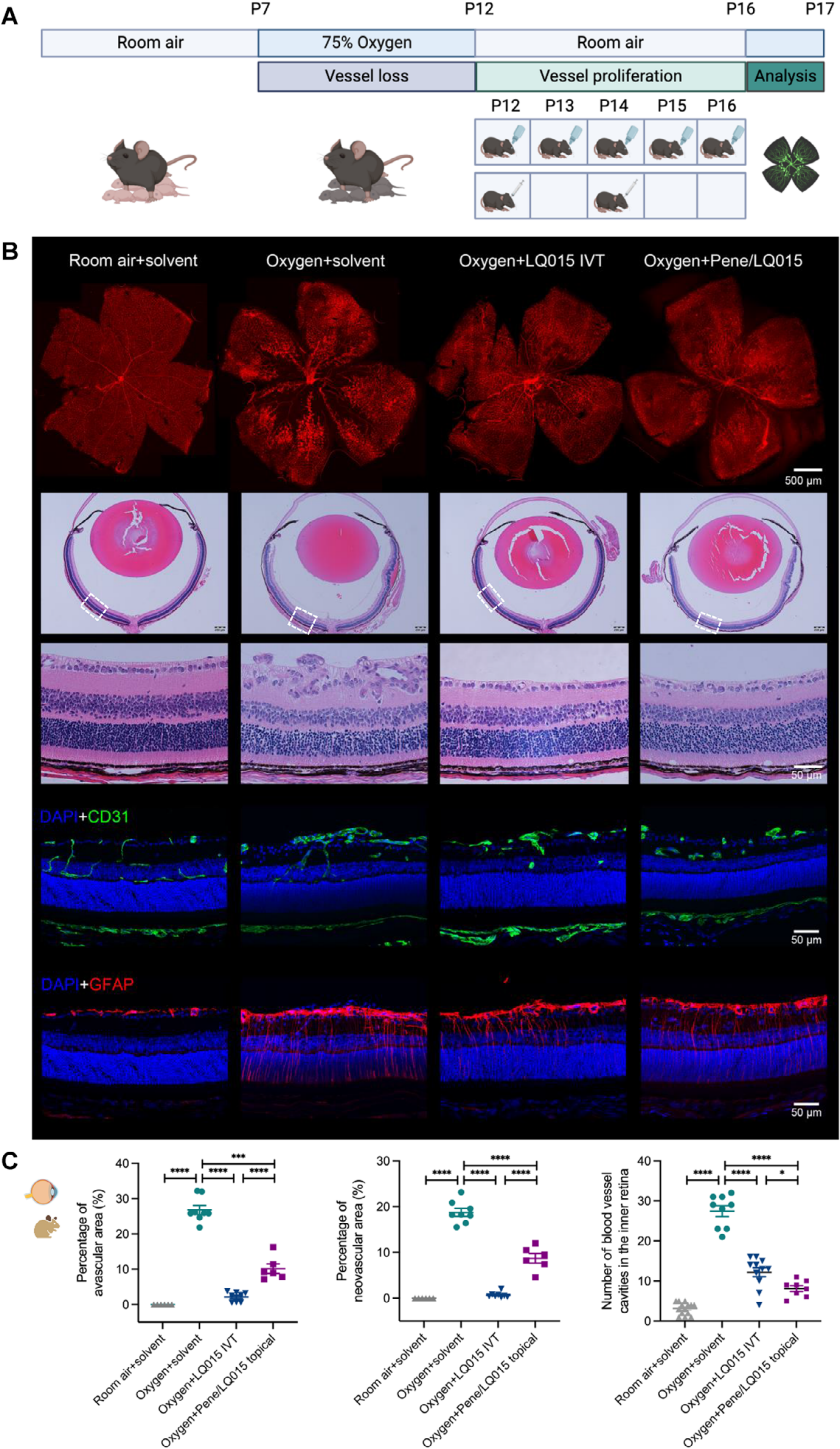
Pene/LQ015 eye drops as a novel angiogenesis inhibitor with potential anti‐angiogenic effects through noninvasive administration. A) The schematic diagram of OIR modeling, administration, and anatomical analysis, created by BioRender.com. B) Representative images of retina flatmounts stained with isolectin B4, sections stained with hematoxylin and eosin, and immunofluorescence at P17 showed that both intravitreal LQ015 injection and topical Pene/LQ015 eye drops inhibited pathological retinal neovascularization and improved avascular regions. C) Avascular area, neovascular area and tufts were reduced in both topical Pene/LQ015 eye drops and intravitreal LQ015 injection treatment groups (*n* = 6–14). IVT: Intravitreal injection; DAPI: 4’,6‐Diamidino‐2‐phenylindole; CD31: Cluster of Differentiation 31; GFAP: Glial Fibrillary Acidic Protein. Data are presented as means ± SEM and analyzed with one‐way ANOVA, followed by Tukey's multiple comparison in (C). ^*^ *p* < 0.05; ^***^ *p* < 0.001; ^****^ *p* < 0.0001.

In the C57BL/6J mouse oxygen‐induced retinopathy (OIR) model, topical administration of Pene/LQ015 eye drops (3:1 ratio, 3 µL per eye, 3 times per day, LQ015 20 mg mL^−1^) or equal volume solvent were given from postnatal day 12 (P12) to P16, while intravitreal injection of 1 µL LQ015 (10 mg mL^−1^) were given on P12 and P14 (**Figure** **5**A). Analysis of the vasculature labeled by isolectin B4 in retinal flatmounts, hematoxylin and eosin‐stained retinal sections, and immunofluorescence‐stained frozen sections on P17 revealed that the progression of pathologic retinal neovascularization and avascular regions was suppressed by the topical application of Pene/LQ015 eye drops or intravitreal injection of LQ015 (Figure 5B). Quantitative analysis demonstrated a significant reduction in the avascular area (*p* < 0.001) and pathologic neovascular changes (*p* < 0.0001) in the OIR model following treatment with Pene/LQ015 eye drops compared to the solvent group (Figure 5C).

### 2.9 In Vivo Efficacy and Pharmacodynamic Study of Pene/LQ015 Eye Drops in Cynomolgus Monkey Laser Induced CNV Model

**Figure 6:**
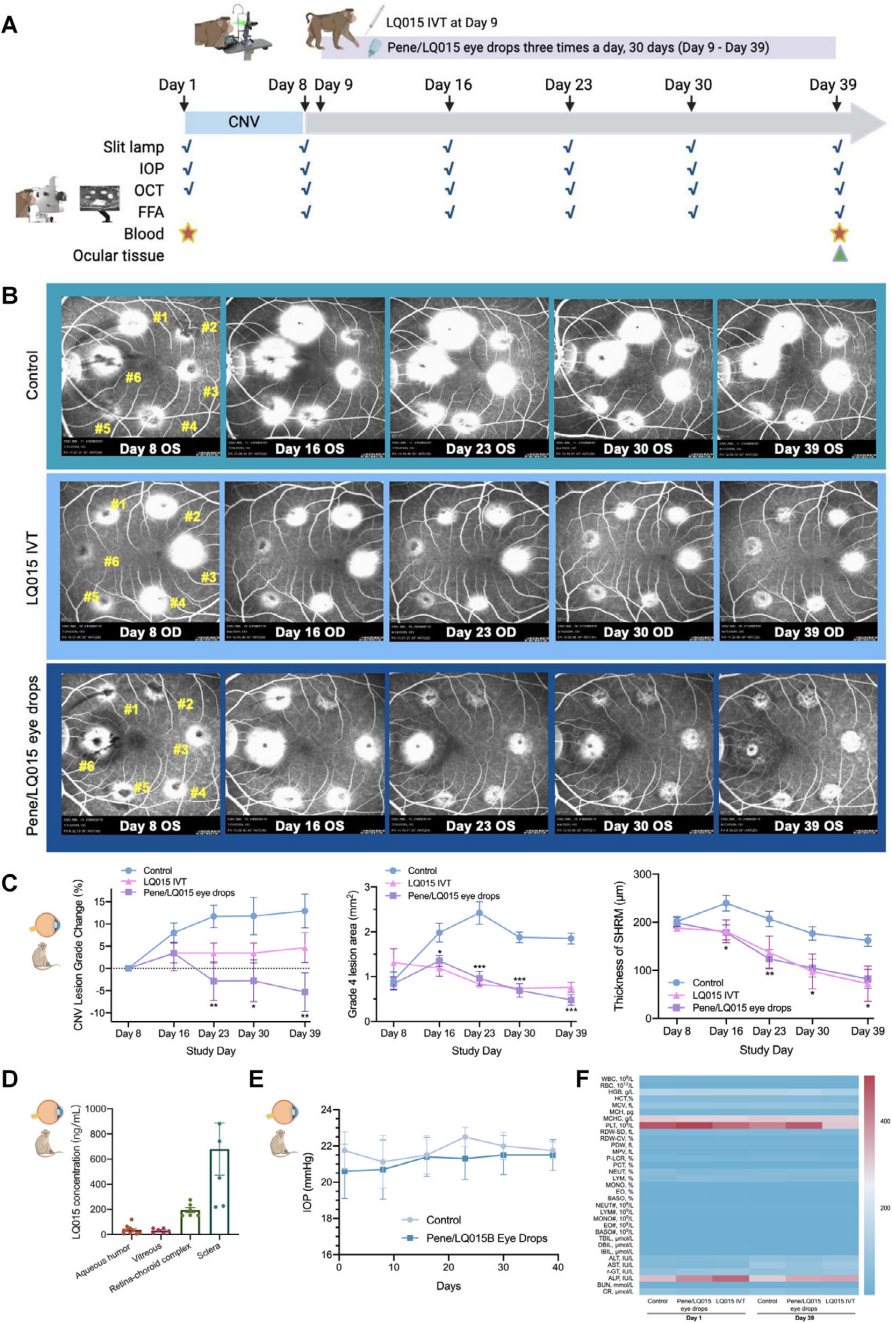
Pene/LQ015 eye drops for continuous inhibition of pathological retinal and choroidal neovascularization in cynomolgus monkey. A) The schematic representation of the modeling process, administration procedure, examination method, and distribution concentration of LQ015 in the laser‐induced CNV cynomolgus monkey model, created by BioRender.com. B) The representative fundus fluorescein angiography images of laser‐induced CNV model of Cynomologus monkey from the solvent topical administration group (*n* = 8 eyes), LQ015 intravitreal injection group (4 mg/50 µL, single dose, *n* = 4 eyes), and Pene/LQ015 topical administration group (LQ015: 1.2 mg/30 µL, three times a day for 30 consecutive days, *n* = 10 eyes) on Day 8 (before administration/intravitreal injection), 16, 23, 30 and 39. C) The alterations in CNV lesion grade and Grade 4 lesion area in each group on Days 16, 23, 30, and 39 in comparison to Day 8. Topical administration of Pene/LQ015 led to a noteworthy decrease in CNV lesion grade and an improvement in CNV leakage area. Compared to the control group, Pene/LQ015 eye drops demonstrated a significant reduction in SHRM. D) The distribution of LQ015 in various ocular tissues 1 h after the last topical application of Pene/LQ015 eye drops over a 30‐day period in cynomolgus monkeys. The concentrations of LQ015 in the aqueous humor, vitreous, retina‐choroidal complex, and sclera were found to be 36.14 ± 35.12, 30.27 ± 17.18, 193.8 ± 51.68, and 679.6 ± 507.6 ng mL^−1^, respectively. E) IOP of the control group and Pene/LQ015 eye drop group on Day 1, 8, 16, 23, 30, and 39 in the laser‐induced CNV model of Cynomolgus monkey. F) Safety evaluation and blood test results of different groups on Day 1 and Day 39 in the laser‐induced CNV model of Cynomolgus monkey. CNV, choroidal neovascularization; SHRM: subretinal hyperreflective material thickness; IOP, intraocular pressure. Data are presented as means ± SEM and analyzed with one‐way ANOVA, followed by Tukey's multiple comparison in (C, D, E). ^*^ *p* < 0.05; ^**^ *p* < 0.01; ^***^ *p* < 0.001.

Further evaluation in a laser‐induced choroidal neovascularization (CNV) cynomolgus monkey model demonstrated the long‐term efficacy of Pene/LQ015 eye drops (**Figure** **6**A). Figure 6B presents representative fundus fluorescein angiography (FFA) images from the solvent topical administration control group, Pene/LQ015 topical administration group, and LQ015 intravitreal injection group on Day 8 (before administration/intravitreal injection), 16, 23, 30, and 39. Daily topical administration of Pene/LQ015 resulted in a decrease in Grade 4 CNV lesion percentage (Figure S3A, Supporting Information), a reduction in average CNV lesion Grade, a notable improvement in Grade 4 CNV leakage area, a decrease in subretinal hyperreflective material thickness (Figure 6C), and retinal thickness (Figure S3B, Supporting Information) compared to controls. While the onset of action was slightly delayed compared to intravitreal injection, continuous daily administration resulted in superior CNV leakage area reduction by Day 39 (Figure 6B).

Quantitative analysis of ocular tissue distribution on Day 39 revealed therapeutic LQ015 concentrations in the retina‐choroid complex (193.8 ± 51.68 ng mL^−1^), exceeding the effective dose threshold,^[^ ^31^ ^]^ along with detectable levels in aqueous humor (36.14 ± 35.12 ng mL^−1^), vitreous (30.27 ± 17.18 ng mL^−1^), and sclera (679.6 ± 507.6 ng mL^−1^) (Figure 6D).

### 2.10 In Vivo Safety Evaluation of Pene/LQ015 Eye Drops

In vivo safety assessments in cynomolgus monkeys with prolonged topical administration (3 times daily for 30 days) induced no detectable abnormalities in behavior, ocular health, or systemic parameters. Comprehensive ophthalmic evaluations, including slit‐lamp microscopy of the anterior segment (conjunctiva, cornea, anterior chamber, iris, and lens) and posterior segment examinations (vitreous, optic disc, macula, retina, and retinal vasculature using a 120D lens or indirect ophthalmoscopy) at Day 1, 8, 16, 23, 30, and 39, revealed no structural alterations. Additionally, intraocular pressure (Figure 6E) and hematological profiles (Figure 6F; Table S1, Supporting Information) remained stable throughout the treatment period, underscoring the formulation's biocompatibility.

To further evaluate potential retinal toxicity, retinal function was assessed in SD rats following 5 days of topical Pene/LQ015 administration. Electroretinography (ERG) demonstrated no significant differences in a‐ or b‐wave amplitudes or morphology between treated and control eyes across all tested responses (rod, maximal, and cone; *p* > 0.05; Figure S4, Supporting Information), confirming the absence of functional impairment.

Together, these data from nonhuman primate and rodent models robustly demonstrate the safety of Pene/LQ015 eye drops for ocular application, with no adverse effects on tissue integrity, physiological metrics, or retinal electrophysiology at selected doses.

## 3 Discussions

Our study demonstrates that LQ015, as a highly specific VEGFA inhibitor, effectively blocks pathological angiogenesis by preventing VEGFA‐VEGFR2 interactions (Figure 2B,D,E,G,H). This inhibition disrupts downstream PI3K/AKT and MAPK/ERK signaling pathways that mediate endothelial cell proliferation, migration, and vascular hyperpermeability.^[^ ^32^ ^]^

The therapeutic potential of LQ015 is significantly enhanced through its innovative formulation with penetratin analog via non‐covalent electrostatic interactions, which facilitates efficient noninvasive intraocular delivery despite the eye's formidable anatomical barriers. The mechanism of ocular penetration for cationic CPPs like our penetratin analog appears to involve multiple pathways. While the exact mechanism remains to be fully elucidated, studies on traditional penetratin (RQIKIWFQNRRMKWKKK) suggest that its transcytosis occurs through both direct transmembrane transport (mediated by electrostatic interactions with negatively charged cell membranes) and endocytic pathways involving caveolae and clathrin‐mediated processes.^[^ ^11^, ^28^ ^]^ Recent findings with cationic CPP R9 further reveal that membrane penetration occurs through electrostatic binding to anionic lipids, inducing membrane fluidization and phase transition from ordered (Lo) to disordered (Ld) lipid domains, enabling translocation without pore formation.^[^ ^23^ ^]^

The non‐covalent delivery strategy of LQ015 and penetratin analog offers several distinct advantages: (1) preservation of the native structure and bioactivity of both components; (2) flexible adjustment of the CPP‐to‐nanobody ratio for optimal formulation; and (3) simple preparation through mixing, facilitating scalable production.^[^ ^33^ ^]^

Our pharmacokinetic studies in rabbits revealed that the achieved LQ015 concentrations via Pene/LQ015 topical administration (3 times daily for 7 consecutive days, retina‐choroid complex: 887.8 ± 135.2 ng mL^−1^ at 1 h; Figure 4B) were comparable to the therapeutic levels of Aflibercept following intravitreal injection in rabbits (1000 ng g^−1^ at 15 days and 80 ng g^−1^ at 30 days postinjection).^[^ ^31^ ^]^ This finding suggests that regular topical application could maintain therapeutic drug levels, potentially serving as an effective adjunct to anti‐VEGF intravitreal injections.

Although in vitro assays confirmed efficient cellular uptake of Pene/LQ015 in both HCECs and ARPE‐19 cells (Figure 3A), the in vivo distribution pattern suggests a predominant conjunctival‐scleral‐choroidal‐retinal transport pathway rather than direct corneal penetration. This discrepancy likely arises from the anatomical complexity of intact ocular tissues, including multicellular barriers and dynamic tear clearance,^[^ ^20^, ^34^ ^]^ which are not fully recapitulated in monolayer cell models. The observed posterior segment drug accumulation in rabbits (Figure 4B) supports transscleral diffusion as the primary route for intraocular delivery,^[^ ^11^, ^20^ ^]^ while preserved HCEC permeability indicates future potential adaptability for cornea or anterior segment applications through enhanced corneal retention with optimized formulation strategies.

Additionally, comparative efficacy studies revealed route‐dependent therapeutic profiles. In the OIR mouse model, intravitreal LQ015 (two doses at P12 and P14) showed superior anti‐angiogenic effects compared to 5‐day topical Pene/LQ015 treatment (Figure 4B,C). Conversely, in laser‐induced CNV in cynomolgus monkeys, 30‐day topical Pene/LQ015 achieved better CNV suppression than single LQ015 intravitreal injection, particularly in CNV lesion grade improvement (Figure 5B,C). This pattern mirrors clinical practice, where intravitreal injections provide a rapidly acting intervention, while topical therapy enables long‐term chronic management. Although topical Pene/LQ015 may take longer to reach therapeutic efficacy compared to direct intravitreal delivery, sustained non‐invasive administration can ultimately achieve comparable therapeutic outcomes.

Notably, the cynomolgus monkey study, designed to simulate clinical practice conditions, provides compelling evidence that Pene/LQ015 eye drops could serve as an adjunctive and complementary treatment to current first‐line anti‐VEGF intravitreal injection therapies (Figure 6). The efficacy profile observed in these preclinical models highlights the potential of topical Pene/LQ015 as a maintenance therapy following initial intravitreal injection treatment.

Future investigations will focus on: optimization of formulation parameters (e.g., dosing frequency, concentration) for different clinical indications through pharmacokinetic/pharmacodynamic modeling; expansion to anterior segment applications including corneal neovascularization through targeted formulation adaptations; exploration of combination therapeutic regimen with existing anti‐VEGF agents to potentially enhance treatment outcomes; clinical translation through IND‐enabling studies and phase I clinical trials to establish human safety and efficacy.

## 4 Conclusion

In conclusion, LQ015 represents a promising next‐generation anti‐VEGFA nanobody therapeutic, characterized by its enhanced efficacy, potent antiproliferative activity, and favorable safety and stability profiles. The innovative formulation of LQ015 with proprietary penetratin analog in eye drop form enables effective noninvasive delivery, achieving sustained therapeutic concentrations in the retina‐choroid complex for continuous neovascular lesion stabilization.

Our findings strongly support the further development of Pene/LQ015 as a transformative therapeutic candidate for various neovascular ocular conditions, including retinal/choroidal vascular diseases, secondary macular edema, iris neovascularization, and neovascular glaucoma. This novel approach offers several significant advantages over current intravitreal anti‐VEGF therapies: 1) it provides a noninvasive alternative to frequent intraocular injections, serving as either a standalone treatment or adjunct therapy that could reduce intravitreal injection frequency; 2) noninvasive eye drops enable relatively stable and sustained therapeutic dosing in target tissues through improved patient adherence.

The Pene/LQ015 eye drop formulation has the potential to revolutionize the treatment paradigm for neovascular fundus diseases, addressing critical unmet needs in long‐term disease management while improving patient quality of life.

## 5 Experimental Section

### Materials and Reagents

The penetratin analog peptide (molecular weight: 2.435 KDa) was provided by Alephoson Biopharmaceuticals Limited (Hong Kong SAR, China) as a gift. HUVEC, HCEC, ARPE19, and HRMEC cells were purchased from the American Type Culture Collection (Maryland, USA) and cryopreserved in the tissue culture laboratory at the Department of Ophthalmology, Shanghai General Hospital (Shanghai, China). Fetal bovine serum and Dulbecco's modified Eagle's medium were purchased from Gibco (Rockville, MD, USA). Cell counting kit‐8 (CCK‐8) was purchased from Beyotime (Shanghai, China). Alexa Fluor 568‐conjugated isolectin B4 was purchased from Molecular Probes (Eugene, OR, USA). If not specifically stated, all other reagents were obtained from Thermo‐Fisher or Sigma‐Aldrich. All chemicals used were of analytical grade.

### Animals

Neonatal C57BL/6J mice with mothers, Sprague‐Dawley rats (0.15 kg), and adult New Zealand rabbits (2.5 kg) were provided by the Shanghai Laboratory Animal Center at the Chinese Academy of Sciences. Rhesus monkeys were provided by the WestChina Frontier PharmaTech Co. Ltd., and cynomolgus monkeys were provided by Guangzhou Huazhen Biotechnology Co., Ltd. The animals were housed in an air‐conditioned room at a 12 h light‐dark cycle with access to food and water ad libitum. The animals were acclimatized to laboratory conditions for 1 week prior to the experiments. All animal experiments were approved by the Medical Ethics Committee of the Shanghai General Hospital, School of Medicine, Shanghai Jiao Tong University (2023AW047), the Institutional Animal Care and Use Committee, Shanghai Institute of Materia Medica (2023‐03‐RJ‐298), and the Department of Pharmaceutics, School of Pharmacy, Fudan University (2022‐03‐YJ‐WG‐13). Animal experiments were conducted in accordance with the NIH Guide for the Care and Use of Laboratory Animals.

### Cytotoxicity of LQ015 and Pene/LQ015 Compounds

The cytotoxicity of LQ015 and Pene/LQ015 compounds was evaluated using the CCK‐8 assay according to the manufacturer's instructions. HCECs and ARPE19 cells were seeded in 96‐well plates at densities of 3 × 10⁴ and 1 × 10⁴ cells per well, respectively, in 100 µL of culture medium, and incubated at 37 °C with 5% CO_2_; for 24 h. Cells were then treated with LQ015 (5 µm), penetratin analog (5 or 15 µm), or Pene:LQ015 mixtures at molar ratios of 1:1 and 3:1, followed by incubation at 37 °C for 24 h. After treatment, the medium was replaced with fresh culture medium, and cells were incubated for an additional 1 h. Subsequently, 10 µL of CCK‐8 reagent was added to each well, and cells were incubated for 4 h. Absorbance was measured at 450 nm using a microplate reader, and cell viability was expressed as relative absorbance. All experiments were performed in triplicate.

### Cellular Uptake Assay

HCECs and ARPE19 cells were digested, passaged, and seeded in 6‐well plates at a density of 3 × 10⁵ cells per mL. After 24 h of incubation, the culture medium was replaced with fresh medium containing 0 or 5 µm Cy5‐labeled LQ015, either alone or in combination with penetratin analog at molar ratios of 1:2 or 1:1. Cells were incubated at 37 °C for 1 or 4 h. Following incubation, cells were washed with 1000 IU mL^−1^ heparin on ice for 20 min to remove extracellularly bound peptides. Cells were then resuspended in 1 mL of PBS per well and immediately analyzed using a high‐content imaging and analysis system (Operetta CLS, PerkinElmer) to quantify the internalization of LQ015‐Cy5.

### Flow Cytometry Analysis

HCECs and ARPE19 cells were digested, passaged, and seeded in 6‐well plates at a density of 3 × 10⁵ cells per mL. After 24 h of incubation, cells were treated with fresh medium containing varying molar ratios of penetratin analog to LQ015 (1:2 or 3:1) and incubated for 1 or 4 h. Cells were then washed with PBS containing 0.02 mg mL^−1^ sodium heparin, harvested, and analyzed using a flow cytometer (CytoFLEX, Beckman Coulter).

### In Vitro Experiments Performed with HRMECs

HRMECs (passages 3‐8) were maintained in Complete Classic Medium (cell systems) supplemented with 10% fetal bovine serum and CultureBoost (cell systems) at 37 °C in a 5% CO_2_ humidified atmosphere, using Attachment Factor™ (cell systems) ‐coated culture dishes.^[^ ^35^ ^]^ For both migration and tube formation assays, cells were divided into six experimental groups: Control, VEGF (25 ng mL^−1^ for migration; 15 ng mL^−1^ for tube formation), VEGF + Aflibercept (0.4 mg mL^−1^), VEGF + LQ015 (5 µm), and VEGF + Pene:LQ015 (1:2 and 3:1 ratios). Migration assay^[^ ^35^, ^36^ ^]^: HRMECs (1 × 10^4^ cells/insert) were seeded into transwell inserts with test compounds. Following 24 h incubation, migrated cells were fixed with 4% paraformaldehyde, stained with 0.1% crystal violet, and quantified by counting cells in five random microscopic fields per insert. Tube formation assay^[^ ^35^ ^]^: Pretreated HRMECs (3 × 10^3^ cells/well) were seeded on growth factor‐reduced Matrigel‐coated µ‐Slide Angiogenesis plates. After 2–4 h of incubation, capillary‐like structures were imaged using phase‐contrast microscopy (Olympus IX73). Total tube length and branch points were analyzed from four representative fields per well using ImageJ software (NIH).

### Pharmacodynamic and Formulation Optimization Study of Pene/LQ015 Eye Drops in Rabbits

New Zealand rabbits were administered eye drops containing LQ015 alone or Pene:LQ015 mixtures at molar ratios of 1:2 or 3:1 (50 µL, 40 mg mL^−1^, 3 times daily) into the conjunctival sac for 7 consecutive days. Peripheral blood was collected at 1, 4, and 12 h after the last administration, and plasma was isolated by centrifuging coagulated blood samples at 14 000 × g for 20 min at 4 °C. Simultaneously, the eyes were enucleated, and the aqueous humor, vitreous humor, and retina‐choroid complex were dissected. The concentration of LQ015 in plasma and ocular tissues was quantified using ELISA. This pharmacodynamic study aimed to identify the optimal molar ratio of penetratin analog to LQ015 for the formulation of Pene/LQ015 eye drops, which were subsequently used in further in vivo experiments.

### Mouse OIR Model

To further assess the antiangiogenic effects of Pene/LQ015 eye drops in vivo, we employed the mouse OIR model.^[^ ^20^, ^37^ ^]^ Neonatal C57BL/6J mice were randomly divided into four groups (*n* = 6–14 per group): 1) room air + solvent eye drops, 2) oxygen + solvent eye drops, 3) oxygen + Pene/LQ015 eye drops, and 4) oxygen + LQ015 intravitreal injection. Only the right eye of each pup was treated to ensure successful modeling. Starting at postnatal day 7 (P7), pups and their mothers were exposed to hyperoxia (75% ± 5% oxygen) for 5 days, followed by room air (21% oxygen) for an additional 5 days. From P12 to P16, Pene/LQ015 eye drops (3 µL per eye, LQ015 20 mg mL^−1^) were administered three times daily, while the control group received solvent. Intravitreal injections of LQ015 (1 µL, 10 mg mL^−1^) were performed on P12 and P14.

At P17, pups were euthanized with pentobarbital, and their eyes were enucleated and fixed in 4% paraformaldehyde. The eyes were then paraffin‐embedded, and sagittal sections (10 µm thickness) were cut through the cornea and parallel to the optic nerve. Hematoxylin‐eosin (H&E) staining was performed to quantify new vessel lumens on the vitreous side of the internal limiting membrane.

For retinal vascular analysis, retinas were dissected, flattened, and fixed in 4% paraformaldehyde for 1 h. After permeabilization with 0.5% Triton X‐100 overnight at 4 °C, retinas were stained with Alexa Fluor 568‐conjugated isolectin B4 to visualize the vasculature. Retinas were flat‐mounted on slides with the photoreceptor side facing down, rinsed in PBS, and covered with SlowFade anti‐fade reagent. Fluorescence images were captured using a Carl Zeiss fluorescence microscope (5× magnification).

For immunohistochemistry, mouse eyeballs were enucleated, and the cornea and lens were removed under a microscope. The remaining tissue was fixed in 4% paraformaldehyde for 30 min, dehydrated in 15% and 30% sucrose gradients, embedded in optimal cutting temperature compound, and sectioned at 12 µm thickness. Sections were air‐dried for 15 min, washed with PBS to remove optimal cutting temperature compound, and blocked with 5% donkey serum, 0.3% Triton X‐100, and 0.02% Proclin 300 in PBS for 1 h at room temperature. Primary antibodies against CD31 (R&D) and GFAP (Dako) were applied overnight at 4 °C, followed by secondary antibodies for 1 h at room temperature. Sections were imaged using a Leica SP8 confocal microscope.

Retinal images were analyzed using Adobe Photoshop CC (Adobe, San Jose, CA, USA) to quantify avascular areas, preretinal neovascularization, and normal vascularization.

### Cynomolgus Monkey Laser‐Induced CNV Model

This study aimed to establish a laser‐induced CNV model in cynomolgus monkeys to evaluate the therapeutic efficacy, ocular irritation, and systemic toxicity of Pene/LQ015 eye drops. The CNV induction protocol was adapted from previously described methods.^[^ ^38^, ^39^, ^40^ ^]^

Briefly, CNV lesions were induced in 15 male cynomolgus monkeys using 532 nm laser photocoagulation. The day of laser treatment was designated as Day 1. Eleven monkeys with successful bilateral CNV induction were randomly assigned to three groups: 1) Pene/LQ015 eye drops (*n* = 5, 10 eyes; 1.2 mg/30 µL, three times daily from Day 8 to Day 39), 2) LQ015 intravitreal injection (*n* = 2, 4 eyes; 4 mg/50 µL on Day 8), and 3) negative control (*n* = 4, 8 eyes; solvent only). FFA, optical coherence tomography (OCT), and intraocular pressure measurements were performed on Days 8, 16, 23, 30, and 39. CNV severity was graded on a 1–4 scale based on FFA, and the fluorescence leakage area of Grade 4 lesions was quantified. Retinal thickness and subretinal hyperreflective material were measured using OCT.

Blood samples were collected before and after the first and last treatments for hematological and biochemical analyses. Ocular irritation and systemic adverse reactions were monitored throughout the study. On Day 39, monkeys in the Pene/LQ015 eye drop group were euthanized for histopathological evaluation to assess treatment efficacy and potential toxicities.

### ERG Experiment

To evaluate the functional changes in the neural retina, flash ERG was conducted before and after treatment in solvent control group, LQ015 intravitreal injection group (20 mg mL^−1^), and Pene/LQ015 topical administration group (3 times daily, 5 days; LQ015 10 mg mL^−1^), as previously described.^[^ ^20^, ^41^ ^]^ Scotopic and photopic ERGs were performed on all SD rats at baseline (Day 0) and Day 6. The dark‐adapted scotopic response (rod response; 0.01 cd s m^−^^2^), scotopic flash response (maximum response, cone and rod; 3.0 cd s m^−^^2^), and light‐adapted photopic response (cone response; 10.0 cd s m^−^^2^) were recorded, and the a‐ and b‐wave amplitudes were measured (*n* = 6 per group).

### Statistical Analysis

Data were presented as means ± standard error of the mean (SEM). ELISA measurements were performed in duplicate (*n* = 2), and other in vitro or in vivo experiments were repeated at least three times independently (*n* ≥ 3). For ELISA assays, data were log‐transformed to approximate a normal distribution and to stabilize variance. All datasets were checked for outliers and normality prior to statistical analysis. Statistical analyses were performed using one‐way analysis of variance (ANOVA), followed by Tukey's multiple comparison test (GraphPad Prism 10.0, GraphPad Software, USA; SPSS 22.0, Statistical Software, USA). A *p* value < 0.05 was considered statistically significant.

The methods of screening and expression of anti‐VEGF Nbs, the molecular biological activity of LQ015, the pharmacodynamic, safety, and efficacy evaluation study of LQ015 intravitreal injection are summarized in the Supporting Information.

## Conflict of Interest

Y.W. and M.Z. are coinventors on the patent application for “Anti‐VEGF single domain antibodies and their applications” (PCT/CN 110452297 B). B.L. and G.W. are coinventors on the patent application for “polypeptide eye absorption enhancer and use thereof” (US11213591B2, US11826431B2, and CN108976288B). The authors declare no competing interests.

## Author Contributions

C.C., M.Z., X.F., K.L., and K.J. contributed equally to this work. X.X., G.W., Y.W., B.L., and C.C. conceived and designed the project. C.C. and M.Z. wrote the original manuscript. C.C., M.Z., X.F., K.L., and K.J. conducted the experiments and analyzed data. Y.Z. and H.Z. participated part of the experiments. X.Q. helped design part of the experiment. X.X., K.L., G.W., Y.W., and B.L. supervised the project and revised the manuscript. All authors have read and approved the article.

## Supporting information



Supporting Information

## Data Availability

The data that support the findings of this study are available from the corresponding authors upon reasonable request.
