# Supplementary material for: Continuous Suppression of Pathological Retinal and Choroidal Neovascularization in Cynomolgus Monkeys via Noninvasive Ophthalmic Delivery of a Novel Anti‐VEGFA Nanobody and Proprietary Penetratin Analog Formulation
Source: Adv Sci (Weinh). 2025 Oct 21;13(1):e04660. doi: 10.1002/advs.202504660 (PMC12767097; doi:10.1002/advs.202504660)
Supplement: Supplementary file 1 — Supporting Information [file ADVS-13-e04660-s001.docx]

**Supporting Information**

**Continuous Suppression of Pathological Retinal and Choroidal Neovascularization in Cynomolgus Monkeys via Noninvasive Ophthalmic Delivery of a Novel Anti-VEGFA Nanobody and Proprietary Penetratin Analog Formulation**

**This file includes:**

**1. Supplementary Figures S1 to S4**

**2. Supplementary Table S1**

**3. Supplementary Materials and Methods**

**1. Supplementary figures**

**
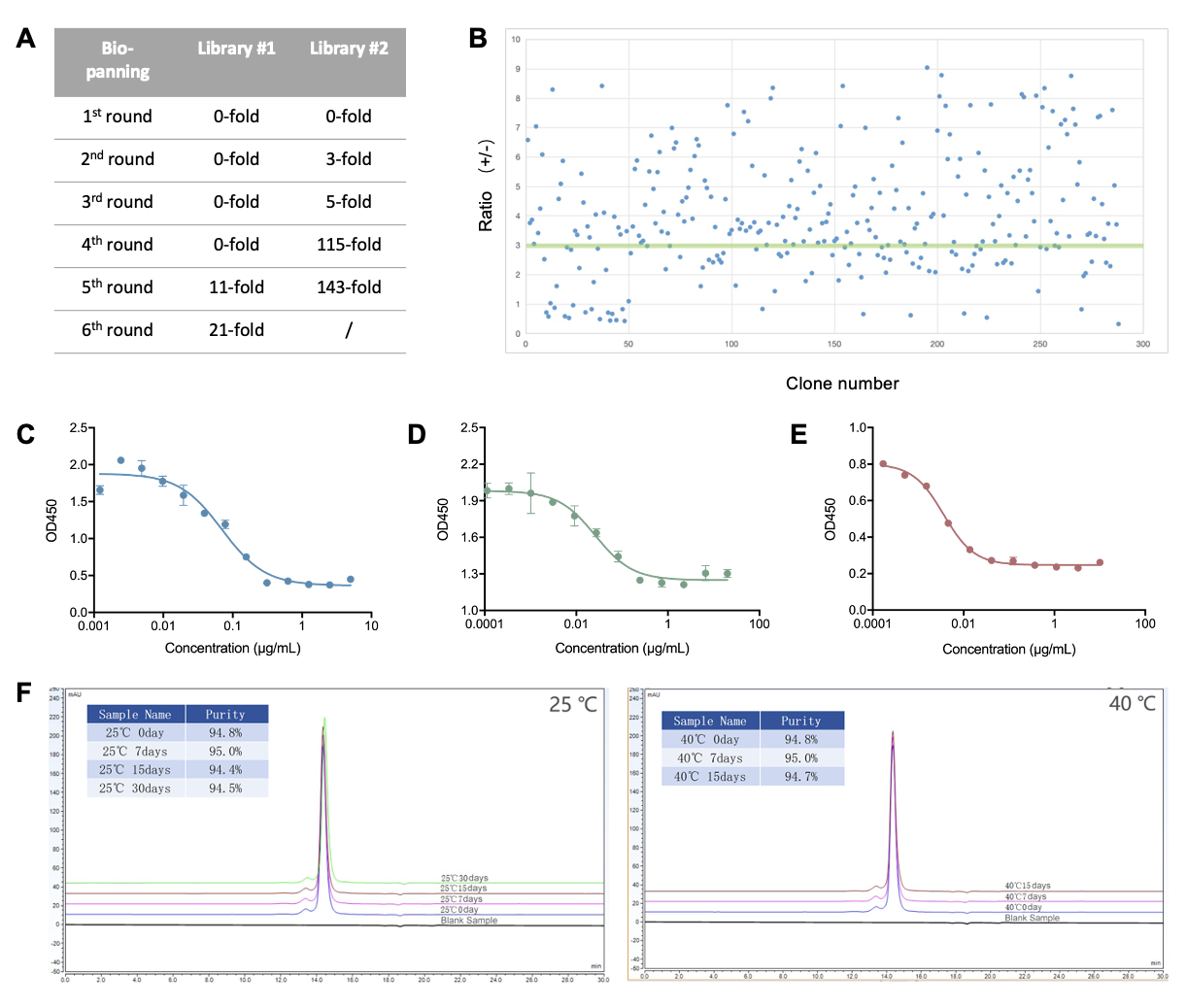
**

**Figure S1.** (A) The screening process was repeated multiple times until phage clones were enriched. (B) The scatter plot shows that a positive clone was determined when the OD_450_ of the screening antigen was more than 3 times greater than the OD_450_ of the negative control. (C-E) LQ015 blocked VEGF/VEGFR2 interaction in multiple genera: (C) rabbit (IC_50_ _rabbit_ = 0.026 μg/mL), (D) rat (IC_50_ _rat_ = 0.070 μg/mL), (E) mouse (IC_50_ _mouse_ = 0.0036 μg/mL). (F) The stability and purity of the LQ015 nanobody at 25℃ and 40℃ was determined through HPLC analysis. Data are presented as means ± SEM in (C, D, E).

**
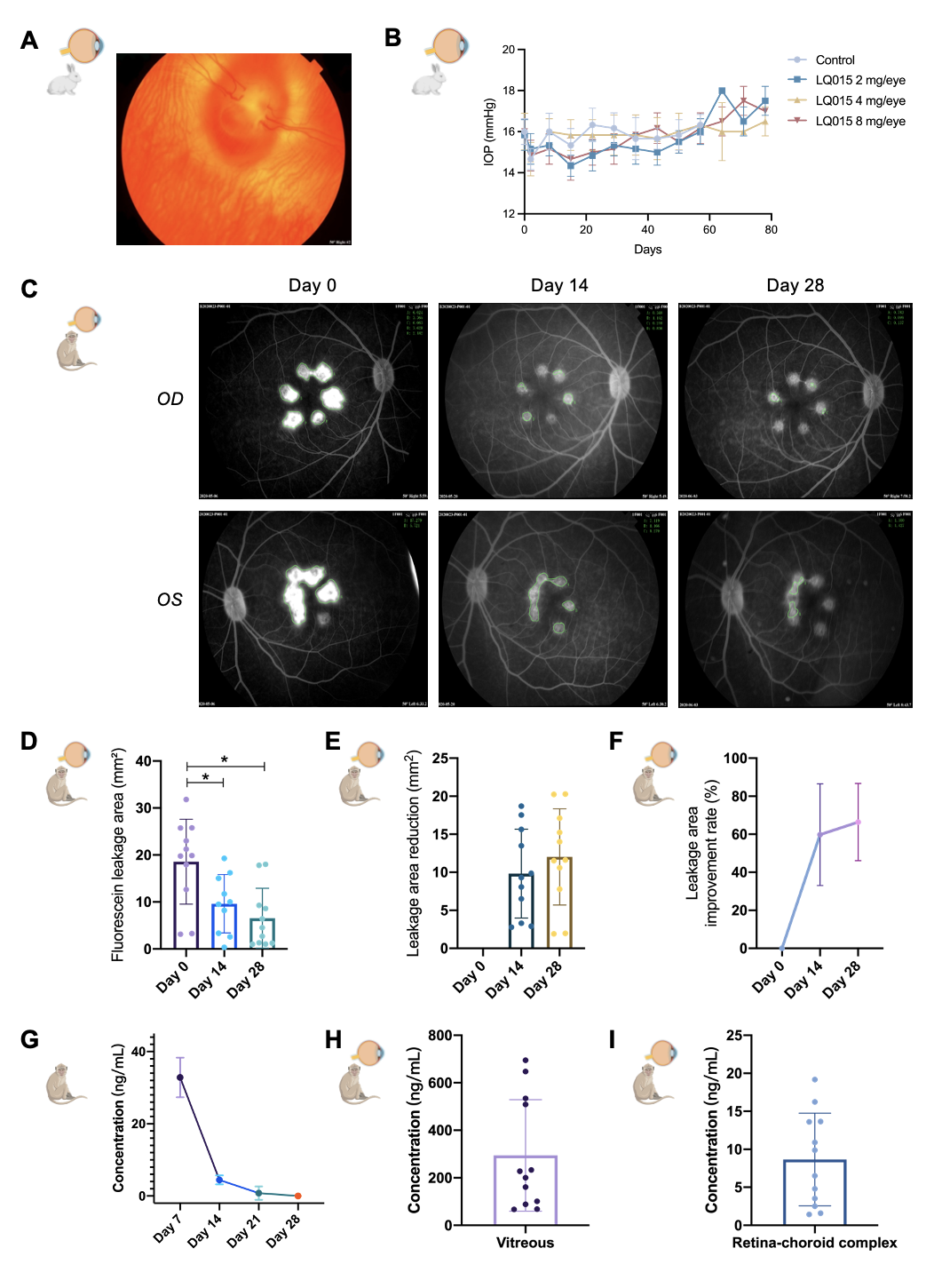
**

**Figure S2.** Efficacy and safety of intravitreal injection of LQ015. (A) Fundus photograph of New Zealand rabbits showed good safety of intravitreal injection of LQ015. (B) There was no significant abnormal change in IOP in both eyes of rabbits in each group [Control, LQ015 2 mg/eye (25 μL), 4 mg/eye (50 μL), 8 mg/eye (100 μL); LQ015 concentration: 80 mg/mL; n = 6 eyes per group]. (C) Representative images of fundus fluorescein angiography on Day 0, Day 14, and Day 28 in the laser-induced CNV model after intravitreal injection of LQ015 in rhesus monkeys. (D) On the 14th and 28th days after administration, the leakage area of fluorescein decreased significantly compared to that of before administration. (E) On the 28th day of administration, the leakage area decreased by 12.026 ± 6.327 mm^2^. (F) The improvement rate of leakage on Day 28 was 66.41 ± 20.32 %. (G) The LQ015 drug concentration in serum samples in laser induced CNV model showed a decreasing trend. On the 28th day of administration, trace amounts of LQ015 were detected in one serum sample; no LQ015 was detected in other serum samples after 21 days of administration. (H & I) A high concentration of LQ015 was detected in the vitreous and retinal-choroid complexes of six rhesus monkeys (n = 11 eyes) on Day 29. The vitreous concentration of LQ015 was significantly higher than that in the retinal-choroid complexes. Data are presented as means ± SEM in (B, D-I) and analyzed with one-way ANOVA, followed by Tukey's multiple comparison in (D). ^*^*p* < 0.05.


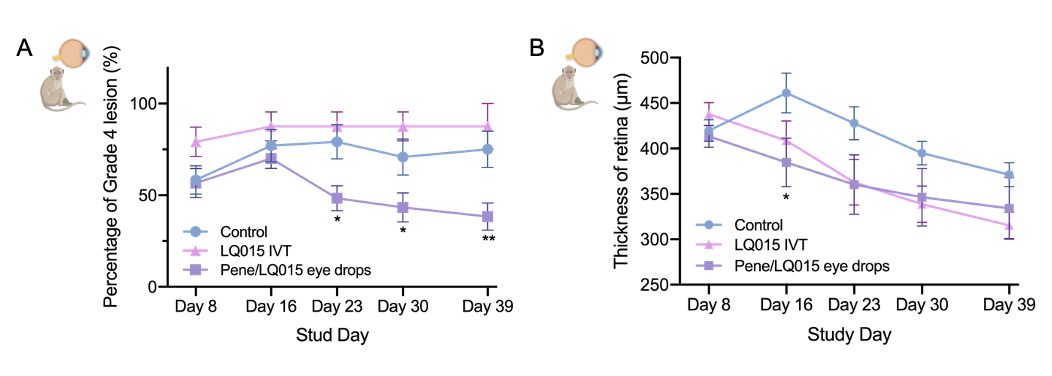


**Figure S3.** In comparison to solvent topical administration control group (n = 8 eyes), the topical administration of Pene/LQ015 eye drops (n = 10 eyes) demonstrated (A) a notable decrease in Grade 4 CNV lesion percentage, and (B) a decrease in the retinal thickness in laser-induced CNV model of Cynomolgus monkey. Data are presented as means ± SEM and analyzed with one-way ANOVA, followed by Tukey's multiple comparison in (A, B). ^*^*p* < 0.05; ^**^*p* < 0.01.


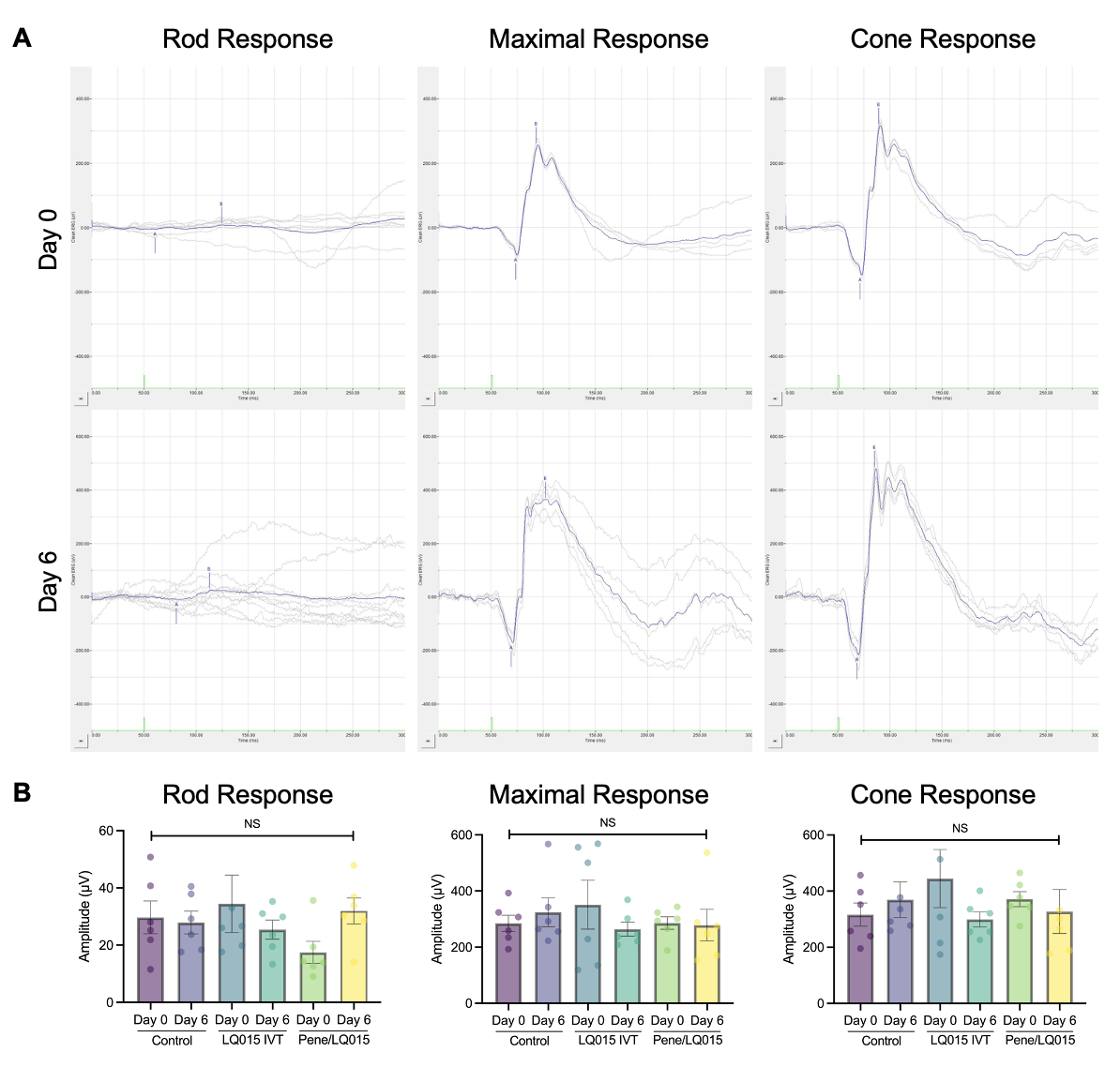


**Figure S4.** Electroretinography (ERG) analysis of retinal function. (A) Representative scotopic ERG traces recorded at 0.01 cd·s/m² (rod response), 3.0 cd·s/m² (maximal response), and 10.0 cd·s/m² (cone response) from Sprague-Dawley rat eyes following topical administration of Pene/LQ015 (twice daily for 5 consecutive days), shown at baseline (Day 0) and Day 6. (B) Quantification of b-wave amplitudes at each stimulus intensity in the control, LQ015 intravitreal injection (IVT), and Pene/LQ015 eye drop groups (n = 6 per group). Data are presented as mean ± SEM. Data are presented as means ± SEM and analyzed with one-way ANOVA, followed by Tukey's multiple comparison in (B). NS: not significant.

**2. Supplementary Table**

**Table S1**. Blood test results of different groups on Day 1 and Day 39 in laser induced CNV model of Cynomolgus monkey.

| **Blood Testing** | **Control** | | | | | **Pene/LQ015 eye drops** | | | | | **LQ015 IVT** | | | | |
| --- | --- | --- | --- | --- | --- | --- | --- | --- | --- | --- | --- | --- | --- | --- | --- |
|  | Day 1 | | Day 39 | | ***p* Value** | Day 1 | | Day 39 | | ***p* Value** | Day 1 | | Day 39 | | ***p* Value** |
|  | Mean | SD | Mean | SD |  | Mean | SD | Mean | SD |  | Mean | SD | Mean | SD |  |
| **WBC, ×10^9^/L** | 17.32 | 2.97 | 10.88 | 1.94 | 0.011 | 14.15 | 4.60 | 11.85 | 2.50 | 0.355 | 14.95 | 1.70 | 11.39 | 3.39 | 0.316 |
| **RBC, ×10^12^/L** | 5.63 | 0.67 | 5.46 | 0.70 | 0.745 | 5.65 | 0.40 | 5.86 | 0.46 | 0.462 | 5.80 | 0.12 | 5.42 | 0.38 | 0.316 |
| **HGB, g/L** | 141.00 | 18.06 | 136.25 | 17.56 | 0.719 | 135.00 | 6.48 | 140.00 | 5.00 | 0.209 | 136.50 | 9.19 | 125.00 | 1.41 | 0.222 |
| **HCT, %** | 46.03 | 4.09 | 44.13 | 4.51 | 0.556 | 45.44 | 2.49 | 46.22 | 1.37 | 0.556 | 46.05 | 2.33 | 40.95 | 1.06 | 0.107 |
| **MCV, fL** | 82.03 | 2.50 | 81.03 | 2.90 | 0.620 | 80.66 | 6.75 | 79.32 | 7.05 | 0.767 | 79.50 | 2.26 | 75.70 | 3.25 | 0.308 |
| **MCH, pg** | 25.05 | 0.75 | 24.93 | 0.29 | 0.766 | 23.98 | 2.07 | 24.02 | 2.12 | 0.977 | 23.55 | 1.06 | 23.15 | 1.34 | 0.772 |
| **MCHC, g/L** | 305.75 | 13.57 | 308.00 | 9.90 | 0.798 | 297.20 | 2.77 | 302.80 | 5.07 | 0.062 | 296.00 | 4.24 | 306.00 | 4.24 | 0.143 |
| **PLT, ×10^9^/L** | 480.50 | 52.85 | 411.50 | 163.47 | 0.452 | 505.60 | 88.08 | 472.60 | 72.30 | 0.535 | 450.00 | 113.14 | 310.50 | 26.16 | 0.231 |
| **RDW-SD, fL** | 37.10 | 2.63 | 35.75 | 2.44 | 0.480 | 38.14 | 2.82 | 37.16 | 1.87 | 0.536 | 37.90 | 0.14 | 36.60 | 2.97 | 0.599 |
| **RDW-CV, %** | 13.10 | 0.67 | 12.78 | 0.44 | 0.448 | 13.74 | 0.61 | 13.64 | 0.90 | 0.842 | 13.80 | 0.28 | 14.10 | 1.70 | 0.828 |
| **PDW, fL** | 15.25 | 0.13 | 15.03 | 0.19 | 0.097 | 15.22 | 0.20 | 15.10 | 0.12 | 0.294 | 15.05 | 0.49 | 15.05 | 0.21 | >0.999 |
| **MPV, fL** | 10.45 | 0.90 | 10.45 | 0.93 | >0.999 | 10.24 | 0.73 | 10.42 | 0.66 | 0.693 | 9.75 | 1.63 | 10.10 | 1.56 | 0.846 |
| **P-LCR, %** | 28.78 | 6.88 | 29.35 | 7.35 | 0.913 | 27.90 | 6.37 | 29.02 | 5.49 | 0.773 | 24.05 | 13.65 | 27.55 | 13.65 | 0.822 |
| **PCT, %** | 0.50 | 0.06 | 0.44 | 0.18 | 0.518 | 0.52 | 0.10 | 0.49 | 0.08 | 0.646 | 0.43 | 0.04 | 0.32 | 0.08 | 0.208 |
| **NEUT, %** | 52.68 | 6.19 | 43.78 | 10.96 | 0.207 | 35.48 | 16.10 | 48.96 | 7.89 | 0.131 | 40.95 | 2.19 | 50.65 | 25.24 | 0.642 |
| **LYM, %** | 40.08 | 4.88 | 48.78 | 9.53 | 0.155 | 55.44 | 14.44 | 43.04 | 7.73 | 0.129 | 49.75 | 7.14 | 40.30 | 23.62 | 0.642 |
| **MONO, %** | 4.93 | 0.90 | 5.35 | 1.87 | 0.697 | 6.00 | 2.15 | 4.72 | 0.67 | 0.240 | 7.15 | 2.90 | 6.25 | 1.48 | 0.734 |
| **EO, %** | 2.18 | 1.94 | 1.95 | 1.44 | 0.858 | 2.86 | 1.74 | 3.10 | 0.89 | 0.791 | 1.90 | 1.98 | 2.60 | 0.00 | 0.667 |
| **BASO, %** | 0.15 | 0.06 | 0.15 | 0.06 | >0.999 | 0.22 | 0.13 | 0.18 | 0.11 | 0.614 | 0.25 | 0.07 | 0.20 | 0.14 | 0.698 |
| **NEUT#, ×10^9^/L** | 9.18 | 2.29 | 4.63 | 0.78 | 0.009 | 5.12 | 2.49 | 5.86 | 1.75 | 0.604 | 6.11 | 0.38 | 5.35 | 1.17 | 0.471 |
| **LYM#, ×10^9^/L** | 6.88 | 1.02 | 5.39 | 1.84 | 0.207 | 7.74 | 3.32 | 5.05 | 1.14 | 0.125 | 7.50 | 1.90 | 5.00 | 4.07 | 0.513 |
| **MONO#, ×10^9^/L** | 0.84 | 0.16 | 0.61 | 0.26 | 0.171 | 0.82 | 0.36 | 0.56 | 0.12 | 0.155 | 1.04 | 0.31 | 0.73 | 0.38 | 0.467 |
| **EO#, ×10^9^/L** | 0.39 | 0.39 | 0.23 | 0.20 | 0.482 | 0.43 | 0.34 | 0.37 | 0.13 | 0.695 | 0.27 | 0.27 | 0.30 | 0.09 | 0.912 |
| **BASO#, ×10^9^/L** | 0.03 | 0.01 | 0.02 | 0.01 | 0.356 | 0.03 | 0.01 | 0.02 | 0.01 | 0.347 | 0.04 | 0.01 | 0.03 | 0.02 | 0.592 |
| **TBIL, μmol/L** | 2.48 | 1.07 | 3.63 | 0.92 | 0.153 | 1.41 | 0.38 | 2.21 | 1.15 | 0.180 | 1.18 | 0.23 | 3.52 | 1.02 | 0.087 |
| **DBIL, μmol/L** | 1.18 | 0.60 | 1.60 | 0.31 | 0.263 | 0.73 | 0.15 | 0.96 | 0.36 | 0.243 | 0.65 | 0.07 | 1.46 | 0.30 | 0.064 |
| **IBIL, μmol/L** | 1.33 | 0.48 | 2.05 | 0.62 | 0.115 | 0.70 | 0.28 | 1.26 | 0.84 | 0.196 | 0.50 | 0.28 | 2.10 | 0.71 | 0.097 |
| **ALT, IU/L** | 43.83 | 17.73 | 71.95 | 23.30 | 0.103 | 43.26 | 6.51 | 61.38 | 13.16 | 0.025 | 48.45 | 17.61 | 76.20 | 17.96 | 0.259 |
| **AST, IU/L** | 39.60 | 11.70 | 98.43 | 28.96 | 0.009 | 33.12 | 5.45 | 71.88 | 44.31 | 0.088 | 39.10 | 19.52 | 99.70 | 43.13 | 0.212 |
| **r-GT, IU/L** | 65.20 | 12.16 | 66.60 | 17.04 | 0.898 | 79.38 | 41.83 | 73.04 | 31.02 | 0.792 | 87.00 | 29.56 | 80.50 | 42.43 | 0.875 |
| **ALP, IU/L** | 326.52 | 31.27 | 302.32 | 28.29 | 0.295 | 402.07 | 162.52 | 358.25 | 121.06 | 0.642 | 456.34 | 104.40 | 363.20 | 129.05 | 0.511 |
| **BUN, mmol/L** | 6.78 | 1.31 | 5.75 | 0.99 | 0.260 | 5.86 | 0.97 | 5.12 | 1.16 | 0.306 | 7.30 | 0.14 | 4.90 | 0.14 | 0.003 |
| **CR, μmol/L** | 74.65 | 12.06 | 73.10 | 7.48 | 0.834 | 61.22 | 8.60 | 61.62 | 9.13 | 0.945 | 58.95 | 0.21 | 54.65 | 3.89 | 0.259 |

CNV, choroidal neovascularization; WBC, White Blood Cell count; RBC, Red Blood Cell count; HGB, Hemoglobin; HCT, Hematocrit; MCV, Mean Corpuscular Volume; MCH, Mean Corpuscular Hemoglobin; MCHC, Mean Corpuscular Hemoglobin Concentration; PLT, Platelet count; RDW-SD, Red Cell Distribution Width - Standard Deviation; RDW-CV, Red Cell Distribution Width - Coefficient of Variation; PDW: Platelet Distribution Width; MPV, Mean Platelet Volume; P-LCR, Platelet Large Cell Ratio; PCT, Plateletcrit; NEUT, Neutrophils; LYM, Lymphocytes; MONO, Monocytes; EO, Eosinophils; BASO, Basophils; NEUT#, Neutrophil count; LYM#, Lymphocyte count; MONO#, Monocyte count; EO#, Eosinophil count; BASO#, Basophil count; TBIL, Total Bilirubin; DBIL, Direct Bilirubin; IBIL, Indirect Bilirubin; ALT, Alanine Aminotransferase; AST, Aspartate Aminotransferase; r-GT, Gamma-Glutamyl Transferase; ALP, Alkaline Phosphatase; BUN, Blood Urea Nitrogen; CR, Creatinine; IVT, intravitreal injection; SD, standard deviation.

**3. Supplementary Materials and Methods**

**Screening and expression of anti-VEGF Nbs**

Immunization: Briefly, the camel was given a series of seven weekly subcutaneous injections in the neck. Each injection consisted of 1 mg of high-purity VEGFA antigen mixed with Freund's complete adjuvant for the first immunization, and with Freund's incomplete adjuvant for the subsequent immunizations.^[1-3]^

Nanoantibody library construction: On day 3 after the last immunization, 100 mL of camel peripheral blood was collected. Peripheral blood mononuclear cells were isolated and total RNA was extracted using an RNA extraction kit. The extracted RNA was then reverse-transcribed into complementary DNA. A two-step PCR method was used to amplify the VHH fragment. In the first round of PCR, the VHH-h-CH2-CH3 fragment, approximately 700 bp in size, was obtained. This fragment served as the template for the second round of PCR, resulting in a VHH fragment of approximately 400 bp. The phage display vector pMECS and the 2nd PCR product fragment were digested using restriction endonucleases Pst I and Not I. Ligation was performed with T4 ligase. The ligated product was transformed via electroporation into TG1 E. coli to construct a Nanobody library. The volume of the library was determined by serially diluting it and spreading it onto a plate, followed by counting the number of single colonies that grew. Additionally, 24 clones were randomly selected from the gradient dilution plates mentioned above for colony PCR to detect the insertion rate of the Nanobody library.

Screening: The obtained camelid nanobody phage display library was used for bio-panning. The VEGFA antigen protein and negative control protein were coated onto a microplate at a concentration of 200 μg/mL and incubated overnight at 4°C. The next day, the blocking solution was added and incubated at room temperature for 2 hours. After blocking, the microplate was washed with PBST solution 10 times. Then, the library phage was added and incubated at room temperature for 1 hour. Following the incubation, the microplate was washed 5 times with PBST to remove non-specific phage. The phage specifically binding to the antigen protein was dissociated using an eluent, and the infected Escherichia coli TG1 cells, which were cultured at 37ºC for 1 hour, were used to propagate the phage for the next round of screening. This screening process was repeated multiple times until phage clones were enriched.

PE-ELISA: 300 single colonies from the enriched phages were randomly selected and inoculated into TB medium containing Amp, then cultured in a 96-well plate. Once reaching the logarithmic phase, IPTG was added for induction and the cultures were incubated at 28 ºC overnight. Subsequently, the crude antibody was obtained using the osmotic shock method. The antibody supernatant transferred to microplate coated with the antigen. After incubation for 1 h at room temperature, the microplate was washed with TBST, and then anti-His HRP antibody was introduced and incubated for 60 min. Subsequently, TMB chromogenic solution was added, and the absorbance value at a wavelength of 450 nm was read using a microplate reader. A positive clone was determined when the OD450 of the screening antigen was more than 3 times greater than the OD450 of the negative control.

**Blocking activity screening for functional anti-VEGF Nbs**

Single domain antibodies that can block the interaction between human VEGFA and VEGFR2 were screened by ELISA. Briefly, VEGFR2 protein was coated on the enzyme label plate (1 μg/mL, 100 μL/well) at 4 ℃ overnight. After 1% BSA blocking for 2 h, the supernatant of clonies or gradient diluted antibody samples mixtured with biotinylated VEGFA protein and then were incubated with the coated VEGFR2 protein at 37 ℃ for 1 h. Finally, SA-HRP were added as the detector and used TMB solution and 2M H_2_SO_4_ to finish the chromogenic reaction.

**Inhibitory effect of candidates on the proliferation of HUVECs**

Briefly, digest the well-growing HUVECs with trypsin, neutralize them completely in the culture medium, wash them with PBS, resuspend them at the concentration of 3 × 10^4^ cells/mL, sub pack them into 96 well plates at 100 μL/well, and culture them at 37 ℃ and 5% CO_2_ for 20 h. Next day, VEGFA and the Nbs were diluted to 100 ng/mL with DMEM of 2% FBS, and added into cells, then co-incubated at 37 ℃ for 2 h. Take out the cell culture plate from the incubator, suck the supernatant, then add 100 μL of the above mixture into the corresponding wells respectively, and incubate at 37 ℃. After 72 h, 10 μL/well of CCK-8 solution were added 2 h. Then measure the absorbance at 450 nm with a microplate reader (BioTek, Synergy LX, USA).

**Construction and expression of humanized bivalent Nb20**

The variable region of the candidate Nb remained unchanged, and the four framework region sequences were humanized. Two humanized Nb20 were constructed into bivalent forms linked by 9GS linker, and then expressed by *Pichia pastoris*. Briefly, construct the nucleotides of bivalent nanobody into pPICZaA vector, and the recombinant plasmid pPICZaA-Nb20-Nb20 (LQ015) was linearized with *Sac*I restriction enzyme and electrotransformed into X-33 competent cells. The electroporation samples are respectively coated on YPD plate containing bleomycin and cultured in 30 ℃ incubator for 3 days. Clonies on the plate were picked into BMGY medium. When the OD reached to 20, the medium was changed to BMMY and added 1% methanol to stimulate the nanobody expression. After continuous induction for 3 days, the supernatant was collected and purified by protein A resin to obtain the bivalent nanobody, which named LQ015.

**Blocking activity and proliferation inhibitory effect of LQ015**

The blocked ELISA and proliferation inhibition method are the same as mentioned above.

**Specificity and cross reactivity of LQ015**

ELISA was used to verify whether the LQ015 could cross react with VEGF family proteins. 1 μg/mL LQ015 was added to the enzyme plate and coated overnight at 4 ℃, 100 μL/well; After washing with PBST, 300 μL 1% BSA were added to each well and sealed at room temperature for 2 h. Then, 100 μL 1 μg/mL biotin-hVEGFA, biotin-hVEGFB, biotin-hVEGFC and biotin-hVEGFD were added and incubated at 37 ℃ for 1 h. The diluted SA-HRP (1:5000 dilution) were added and incubated at 37 ℃ for 1 h. After washing with PBST, add 100 μL TMB chromogenic solution for 10 min and 2M H_2_SO_4_ for 5 min to terminate the reaction. The absorption value under the wavelength of 450 nm were measured by enzyme micro-plate reader.

Similarly, ELISA was used to detect whether LQ015 could cross react with VEGF of other species. 1 μg/mL of LQ015 were coated into the enzyme plate overnight at 4 ℃. After washing with PBST, added 300 μL 1% BSA to each well and seal at room temperature for 2 h. After washing, 100 μL 1μg/mL of biotin-hVEGFA (human), biotin-mVEGF (mouse) and biotin-rVEGFA (rabbit) were respectively incubated for 1 h. The following ELISA steps are the same as above.

**Systemic and ocular safety evaluation study of LQ015 after multiple intravitreal injection (repeated dose) in rabbits**

This experiment consists of four groups, namely the solvent control group and LQ015 25, 50, and 100 µL/eye groups (LQ015 80 mg/mL, equivalent to 2, 4, and 8 mg/eye), with three New Zealand rabbits in each group. Each group of rabbits was given a solvent control substance (LQ015 vehicle) or corresponding volume of LQ015 (80 mg/mL) by intravitreal injection through both eyes, once every 2 weeks, and continuously administered 3 times. The first day of administration is defined as the first day of the experiment.

During the experiment, the general condition of each group of rabbits were observed every day; indirect ophthalmoscopy, slit lamp examination and intraocular pressure measurement were performed before the first administration, on the 2nd, 8th, 15th, 22nd, 29th, 36th, 43rd, 50th, 57th, 64th, 71st, 78th, 85th and 92nd days of the test. On the 57th, 78th, and 93rd days of the experiment, 9 rabbits, 2 rabbits and 1 rabbit were euthanized for gross anatomical observation, respectively.

**Single dose pharmacodynamic study of LQ015 intravitreal injection in rabbit**

After a single dose of LQ015 bilateral intravitreal injected into New Zealand rabbits’ eyes, blood samples and eye tissues were collected to observe the dynamic changes of LQ015 in rabbit blood and eye tissues. Briefly, 24 rabbits (half male and half female) were given a single bilateral intravitreal injection of LQ015 (50 μL/eye, 4 mg/eye). The blood samples, vitreous and retina-choroid complex of rabbits were collected at 1 and 24 h, 3, 8, 15, 30, 45 and 60 days after administration (3 rabbits at each time point). The concentrations of LQ015 in serum and eye tissues were determined by ELISA, and the pharmacokinetic parameters were calculated. The standard curve range of LQ015 is 0.5 ~ 20 ng/mL.

***In vivo* laser-induced choroidal neovascularization (CNV) in rhesus monkey model**

In this experiment, laser was used to damage the Bruch’s membrane of the retina of rhesus monkeys to establish a model of choroid neovascularization. Monkeys weighed 3.0–3.5 kg and aged from 3 to 5 years (5 males and 1 female). Three weeks after the model was made, fundus fluorescence angiography was performed. Serious ocular inflammation after laser-induction, or without grade III or IV of fluorescence leakage in both eyes were excluded. The CNV induction procedures were based on previously published methods.^[4-6]^ Briefly, rhesus monkeys were anesthetized by intravenous injection of 2.5% pentobarbital sodium (25 mg/kg) and 5% ketamine (10mg/kg), and their pupils were dilated using 0.5% tropicamide-phenylephrine ophthalmic solution (Mydrin®; Santen Pharmaceutical, Osaka, Japan), six laser burns (500-700 mW, 50 μm spot, 50 ms duration) were placed around the macula at a distance of 1–2 papilla diameters using a 532 nm laser (Vitra 532 nm, Quantel Medical) through a slip lamp delivery system and the beam directed to the retina with a Volk Centralis diameter (Volt Optical, Mentor, OH). For laser injury, the morphological endpoint was the appearance of a cavitation bubble without hemorrhage, which was a sign of Bruch’s membrane disruption and will lead to the formation of CNV.

Six rhesus monkeys (one monkey's left eye model was unsuccessful, so not included in the observation, and the remaining 11 eyes were included) were selected into the group, and 11 eyes were given LQ015 by a single bilateral intravitreal injection (4 mg/eye: 80 mg/mL, 50 μL/eye). The first day of administration is defined as the first day of the experiment.

During the experiment, the general condition of all rhesus monkeys was observed every day. Before modeling, before administration (after laser), and on the 14th and 28th day of administration, fundus fluorescence angiography was performed to observe the percentage of fourth grade fluorescent spots, the reduction in fluorescein leakage area, and the improvement rate. On the 7^th^, 14^th^, 21^st^ and 28^th^ day of administration, venous blood of lower limbs was collected. On the 29^th^ day of administration, all rhesus monkeys were dissected. Retina choroid complex and vitreous homogenate of both eyes were taken, and LQ015 drug concentration was detected.

CNV was rated on a 1 to 4 grades. Grade 1 indicates no hyperfluorescence, grade 2 indicates lesions exhibiting hyperfluorescence early or mid-transit with late leakage, grade 3 indicates lesions showing hyperfluorescence without leakage, grade 4 indicates lesions showing bright hyperfluorescence early or mild-transit with late leakage extending beyond the borders of the burned area. Grade 3 and 4 of fluorescein leakage spots were set as ROIs (region of interest).^3^ The area of ROI was measured by tracing the borders of fluorescence leakage using Image J software (Wayne Rasband, Research Service Branch, NIH, Bethesda, Maryland, US) with a fixed calibration.

**References**

1. P. C. Fridy, Y. Li, S. Keegan, M. K. Thompson, I. Nudelman, J. F. Scheid, et al., “A robust pipeline for rapid production of versatile nanobody repertoires,” *Nat. Methods* **2014**, 11, 1253–60.
2. Y. Wouters, T. Jaspers, B. De Strooper, and M. Dewilde, “Identification and in vivo characterization of a brain-penetrating nanobody,” *Fluids Barriers CNS* **2020**, 17, 62.
3. M. Liu, L. Li, D. Jin, and Y. Liu, “Nanobody—a versatile tool for cancer diagnosis and therapeutics,” Wiley Interdiscip. Rev. Nanomed. Nanobiotechnol. **2021**, 13, e1697.
4. O. Olvera-Montaño, L. Baiza-Duran, J. D. Quintana-Hau, M. G. Quiñonez-Alvarado, W. Zeng, L. Gong, et al., “Comparing the efficacy of an anti-human VEGF-A neutralizing antibody versus bevacizumab on a laser-induced choroidal neovascularization (CNV) rhesus monkey model,” Drug Des. Devel. Ther. **2019**, 13, 3813–3821.
5. V. Lambert, J. Lecomte, S. Hansen, S. Blacher, M. L. Gonzalez, I. Struman, et al., “Laser-induced choroidal neovascularization model to study age-related macular degeneration in mice,” Nat. Protoc. **2013**, 8, 2197–2211.
6. K. Lai, Y. Li, L. Li, Y. Gong, C. Huang, Y. Zhang, et al., “Intravitreal injection of triptolide attenuates subretinal fibrosis in laser-induced murine model,” Phytomedicine **2021**, 93, 153747.
